# Comparison of extracellular vesicle isolation methods reveals method-dependent protein and miRNA profiles in saliva

**DOI:** 10.20517/evcna.2025.176

**Published:** 2026-06-10

**Authors:** Elodie Simphor, Jérémy Boulestreau, Romane Marchal, Thi Nhu Ngoc Van, Franck Molina, Malik Kahli

**Affiliations:** ^1^Sys2Diag UMR9005 CNRS - ALCEN, Cap Gamma, Parc Euromédecine, Montpellier 34184, France.; ^2^SkillCell, Montpellier 34184, France.

**Keywords:** Salivary extracellular vesicles, EV isolation methods, miRNA profiling, proteomics, biomarker discovery

## Abstract

**Aim:** Salivary extracellular vesicles (EVs) are promising non-invasive biomarkers for disease diagnosis and monitoring, reflecting both systemic and local physiological states. However, the diversity of EV isolation protocols and the possibility that each method enriches distinct EV subpopulations remain major barriers to reproducibility and data comparability. This study aimed to evaluate the impact of different EV isolation methods on the yield, purity, and molecular composition of salivary EVs from healthy volunteers, thereby clarifying how these methods affect the potential use of salivary EVs as a non-invasive biomarker source for disease diagnosis and monitoring.

**Methods:** We conducted a comprehensive comparison of three EV isolation methods - ultracentrifugation (UC), polyethylene glycol (PEG)-based co-precipitation (Q), and immunoaffinity capture (M) - to evaluate their impact on EV yield, purity, and molecular composition. Salivary EVs from healthy volunteers were analyzed using proteomic and small RNA sequencing approaches.

**Results:** Principal component analysis revealed clear isolation method-dependent clustering, where M-derived EVs displayed the most distinct profile. The UC and Q methods yielded broader proteomic repertoires with higher total protein content, while M-isolated EVs exhibited greater purity and enrichment of trafficking- and lysosome-associated proteins. Among the 731 microRNAs (miRNAs) analyzed, 28 were consistently altered across methods and 65 uniquely enriched in M isolates. Reverse transcription quantitative polymerase chain reaction (RT-qPCR) confirmed the key directional trends. These 93 method-dependent miRNAs were predicted to target genes associated with synaptic structure and neurodegenerative pathways.

**Conclusion:** These findings show that EV isolation methodology profoundly influences the cargo composition of salivary EVs and suggest that M can isolate specific EV populations that may better meet diagnostic requirements.

## INTRODUCTION

Extracellular vesicles (EVs) are membrane-bound particles released by most cell types and play key roles in intercellular communication^[1]^. EVs include exosomes, microvesicles (ectosomes), and apoptotic bodies, which differ in size and biogenesis but share the capacity to transfer molecular cargo [such as proteins, lipids, metabolites, and RNA species including microRNAs (miRNAs)] to recipient cells^[2]^. This exchange influences physiological processes, including immune responses^[3]^, cell proliferation^[4]^, and tissue repair^[5]^, and contributes to pathological mechanisms such as cancer progression^[6]^ and neurodegeneration^[7]^.

The biomedical potential of EVs has attracted growing interest, particularly for their use as biomarkers. EV cargo reflects the physiological state of their cells of origin^[2]^, positioning EVs as candidates for so-called “liquid biopsies”, minimally invasive assays that can provide diagnostic, prognostic, and monitoring information^[8,9]^. EVs have been successfully isolated from blood^[10]^, urine^[11]^, cerebrospinal fluid (CSF)^[12]^, and saliva^[13]^. Among these biofluids, saliva is especially promising due to its ease of collection, non-invasive nature, and reduced risk of blood contamination. Salivary EVs may provide insights into both local and systemic health conditions, including oral cancer^[14]^, Sjögren’s syndrome^[15,16]^, and neurodegenerative disorders such as Alzheimer’s disease^[17]^ and Parkinson’s disease^[18,19]^. Proteomic and transcriptomic changes in salivary EVs have already been linked to early pathological alterations, underscoring their diagnostic potential^[16,19,20]^.

Despite these advantages, isolating EVs from saliva presents unique challenges. Saliva contains abundant proteins, mucins, enzymes, bacterial vesicles, and other contaminants that can interfere with EV isolation and compromise sample purity^[21]^. Moreover, EV populations are heterogeneous, varying in size and cargo composition, which complicates reproducibility across studies.

Several methods are commonly employed to isolate EVs, each offering distinct advantages and limitations. Ultracentrifugation (UC), historically regarded as the gold standard, separates vesicles based on size and density. However, this method is labor-intensive, requires specialized equipment, and may co-isolate protein aggregates and non-vesicular particles^[22,23]^. Precipitation approaches, such as polyethylene glycol (PEG)-based co-precipitation (Q), are scalable and user-friendly but often capture soluble proteins and lipoproteins, potentially compromising downstream analyses^[24-26]^. Immunoaffinity capture (M), which targets tetraspanins such as CD9, CD63, and CD81, provides high specificity but typically yields fewer vesicles and may limit recovery to selected EV subpopulations^[27]^. Emerging affinity-based platforms, such as the amphiphile-dendrimer supramolecular probe (ADSP) array, offer a high-throughput alternative by leveraging chemical affinity to capture EVs via their phospholipid bilayers, thereby enabling *in situ* protein analysis and glycosylation profiling^[28,29]^. This approach addresses some limitations of traditional methods by enhancing sensitivity and reproducibility, particularly for clinical samples. Nevertheless, each method involves trade-offs among yield, purity, and scalability, and the optimal approach depends on the intended downstream application. Whether the goal is molecular cargo characterization (e.g., proteomic, transcriptomic, or lipidomic profiling), evaluation of EV functionality or biodistribution, or biomarker discovery in clinical contexts, the choice of isolation method is critical.

Proteomic and miRNA analyses are central to understanding EV composition and function, as well as their potential as biomarkers. Proteomics enables the identification of EV-associated proteins, such as membrane receptors, signaling molecules, and cytoskeletal components, which reflect the cellular origin and biological activity. However, proteomic profiles can be influenced by co-isolated non-vesicular proteins, making sample purity a critical determinant of data interpretability^[26]^. Similarly, EV-associated miRNAs are remarkably stable and clinically informative^[30-32]^, but their accurate quantification depends on efficient separation of vesicular and non-vesicular RNA fractions. Although both proteomic and miRNA analyses provide complementary insights into EV biology, how different isolation methods shape these molecular readouts (particularly in saliva) remains poorly characterized. Understanding these effects is essential for improving reproducibility and ensuring that biomarker signals accurately reflect vesicle biology rather than technical bias^[33-35]^.

While systematic evaluations of EV isolation have been conducted for plasma^[35-37]^, urine^[38-40]^, and CSF^[41,42]^, comparable analyses for saliva remain limited. Recent studies have begun to highlight the sensitivity of salivary EV profiles to the isolation method used^[26,43,44]^, but comprehensive multi-omics comparisons are still lacking. Given both the diagnostic promise and biological complexity of saliva, establishing best practices for EV isolation is essential.

In a previous study, we compared UC, Q, and M for salivary EV isolation by evaluating yield, purity, protein markers, selected miRNAs, and the effects of filtration^[45]^. The present study extends the analysis to include comprehensive proteomic and small RNA sequencing of EVs obtained by these methods. By integrating proteomic and transcriptomic readouts, we assess how isolation strategies shape the molecular landscape of salivary EVs. Our goal is to provide practical data that inform method selection, support standardization, and facilitate reproducibility in biomarker discovery and clinical translation.

## METHODS

### Saliva collection and sample processing

**Figure 1:**
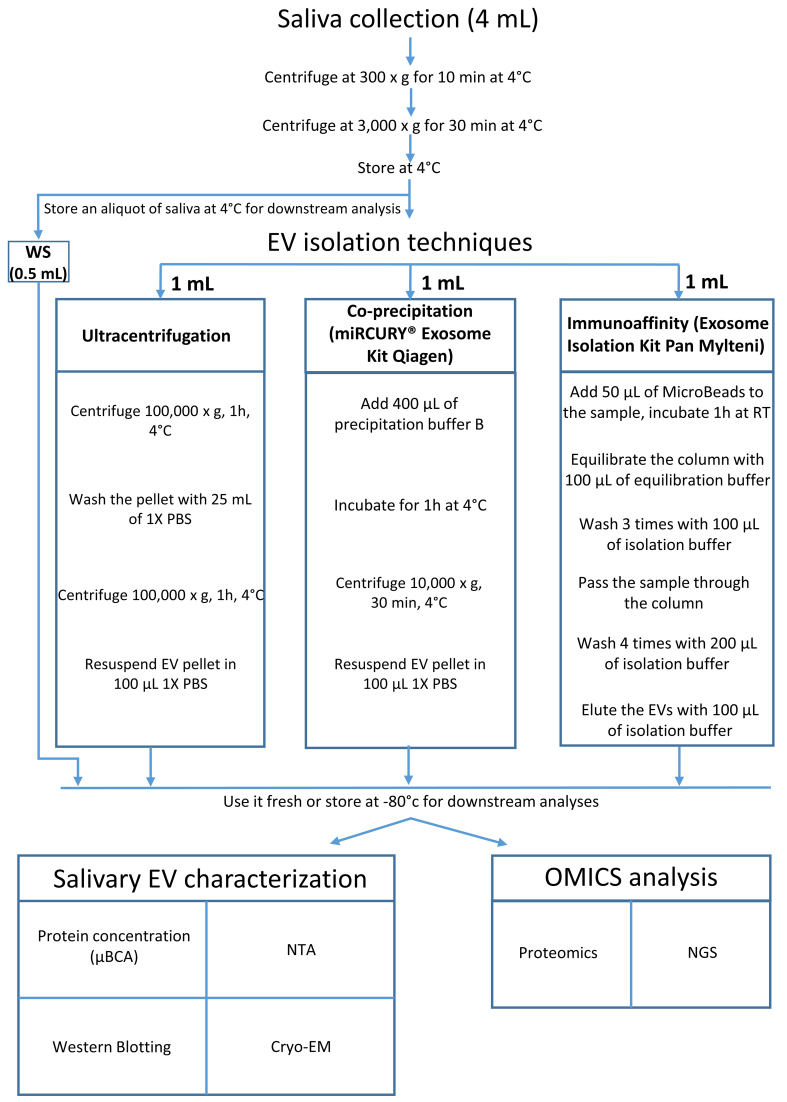
Schematic overview of the experimental workflow for salivary EV isolation and analysis. Unstimulated saliva (4 mL) was collected from healthy donors and sequentially centrifuged at 300 × *g* and 3,000 × *g* to remove cells, large debris, and residual contaminants. The resulting WS was divided into four aliquots: 0.5 mL was retained as the WS control, and 1 mL was allocated to each of the three EV isolation methods: UC, Q, and M. The isolated EVs were subsequently characterized and subjected to proteomic and small RNA sequencing analyses. EV: Extracellular vesicles; WS: whole saliva supernatant; UC: ultracentrifugation; Q: PEG-based co-precipitation; M: immunoaffinity capture; PBS: phosphate-buffered saline; RT: room temperature; NTA: nanoparticle tracking analysis; NGS: next-generation sequencing; EM: electron microscopy; Cryo-EM: cryogenic electron microscopy; μBCA: micro bicinchoninic acid assay.

Unstimulated whole saliva was collected in the morning (9:00-10:00 AM) from nine healthy male volunteers (18-40 years old) who had fasted, refrained from drinking, and avoided smoking for at least 1 h prior to collection. Participants passively drooled into sterile tubes, and a minimum of 4 mL of saliva was collected per individual. Samples showing visible blood contamination were excluded. The collected saliva was placed on ice and processed within 1 h: first centrifuged at 300 × *g* for 10 min at 4 °C to remove cells, followed by 3,000 × *g* for 30 min at 4 °C to eliminate residual debris. The resulting supernatant was used for subsequent EV isolation [Figure 1]. All samples were processed individually (not pooled), and each “n” corresponded to a unique biological replicate. Ethical approval was granted by a French national committee (CPP-NORD OUEST III) on April 21, 2023 (23.00930.000169), and written informed consent was obtained from all participants. The study was registered at www.clinicaltrials.gov (NCT06149351).

### EV isolation and concentration

Three methods were used to isolate EVs from 1 mL of Whole saliva supernatant (WS): UC, Q, and M. Each method was performed in multiple independent batches by different operators to ensure reproducibility and robustness. The procedures followed established protocols^[45]^, and the results demonstrated consistent separation efficiency and EV yield across repetitions, confirming the reliability of the methodology.

UC: WS was diluted in 24 mL of phosphate-buffered saline (PBS) and ultracentrifuged at 100,000 × *g* for 1 h at 4 °C (JXN-30, JA-30.50 Ti rotor, Beckman Coulter). The pellet was resuspended in 10 mL PBS and centrifuged again under the same conditions. The final pellet was resuspended in 100 µL PBS and either analyzed immediately or stored at -80 °C.

Q: EVs were isolated using the miRCURY® Exosome Kit (Qiagen, Cat. #76743). Briefly, 400 µL of precipitation buffer was added to 1 mL of WS, incubated at 4 °C for 1 h, and centrifuged at 10,000 × *g* for 30 min at room temperature. The pellet was resuspended in 100 µL of the provided buffer and either kept on ice or stored at -80 °C.

M: EVs were isolated using the Exosome Isolation Kit Pan, Human (Miltenyi Biotec, Cat. #130-111-572). A total of 50 µL of MicroBeads (anti-CD9, CD63, CD81) was incubated with 1 mL of WS for 1 h at room temperature with agitation. The mixture was then loaded onto a µMACS column, washed, and EVs were eluted in 100 µL of buffer. Bead-only controls were processed in parallel to assess background signals. Eluted EVs were used fresh or stored at -80 °C to preserve integrity.

### EV characterization

EVs were characterized according to the ISEV [Minimal Information for Studies of Extracellular Vesicles (MISEV2023)] guidelines^[46]^. Size distribution and concentration were assessed using Nanoparticle Tracking Analysis (NTA; NanoSight NS300, Malvern Panalytical) in PBS. Measurements (*n* ≥ 3) were acquired with NTA software v3.4 (camera level: 16; detection threshold: 5) and analyzed to determine mean particle size and particle counts.

Cryo- electron microscopy (cryo-EM) was performed by depositing EVs onto glow-discharged Lacey grids, blotting, and vitrifying in liquid ethane using a CP3 cryo-plunge system (Gatan). Grids were imaged on a JEOL 2200FS FEG TEM (200 kV, 4 k × 4 k CCD, 20 eV energy filter) under low-dose conditions. Nanoparticles were classified as vesicles only if they exhibited a clearly visible lipid bilayer and met established size criteria (30-1,000 nm), following MISEV2023 guidelines^[46]^. “Vesicle-in-vesicle” structures, predominantly observed in UC samples, were identified as artifacts resulting from mechanical stress during isolation, whereby smaller vesicles become encapsulated within larger vesicular compartments due to high centrifugal forces and pellet compaction, as previously described^[47-49]^. Non-vesicular particles, such as protein aggregates or antibody-coated beads, were excluded based on the absence of a lipid bilayer.

Protein concentration was measured using the Micro BCA Protein Assay Kit (Thermo Fisher Scientific) at 562 nm (Synergy H1, BioTek) and quantified against bovine serum albumin (BSA) standards (µg/µL). Western blotting for EV marker profiling is described in the following section. All EV samples were either used fresh or stored at -80 °C to preserve integrity.

### Sodium dodecyl sulfate-polyacrylamide gel electrophoresis (SDS-PAGE) and Western blotting

EV samples were lysed in Radio-Immunoprecipitation Assay (RIPA) buffer supplemented with protease inhibitors (Roche) and Laemmli buffer containing 2.5% β-mercaptoethanol. Equal amounts of protein (20 µg) were loaded on 12.5% SDS-polyacrylamide gels and separated at 200 V for 50 min. Proteins were then transferred onto nitrocellulose membranes (Trans-Blot, Bio-Rad) at 90 V for 1 h. Membranes were blocked with 5% skim milk in PBS-Tween 20 (0.1%) for 1 h and incubated overnight at 4 °C with primary antibodies (Abcam): CD9 (ab263019), CD63 (ab134045), Heat Shock Protein 70 (HSP70) (ab181606), Tumor Susceptibility Gene 101 (TSG101) (ab125011) at 1:1,000, and albumin (ab19180) at 1:20,000 dilution. After washing, membranes were incubated with horseradish peroxidase (HRP)-conjugated secondary antibodies (Sigma) for 1 h at room temperature. Signal detection was performed using Clarity Max Chemiluminescence (ECL) substrate (Bio-Rad), and images were acquired with a ChemiDoc MP system (Bio-Rad). Band intensities were quantified using ImageJ.

### Proteomics

Proteomic analysis was performed at the Plateforme de Protéomique Fonctionnelle de Montpellier (FPP) using triplicate samples of WS, UC-, Q-, and M-derived EVs (14.5 µg protein per sample). Proteins were digested using S-Trap^TM^ micro spin columns (Protifi) following the manufacturer’s protocol with minor modifications. Briefly, samples were solubilized in 5% SDS, reduced with 20 mM dithiothreitol (DTT) (10 min, 95 °C), alkylated with 40 mM iodoacetamide (IAA) (30 min, dark), acidified with 1.2% phosphoric acid, and diluted in binding buffer [100 mM triethylammonium bicarbonate (TEABC) in 90% methanol]. Proteins were trapped by centrifugation (4,000 × *g*, 1 min), washed five times, and digested with 1 µg trypsin (Promega) for 2 h at 47 °C. Peptides were eluted, vacuum-dried, and stored for mass spectrometry (MS) analysis.

Peptide separation and analysis were performed via nanoLC-MS/MS using a Q Exactive HF mass spectrometer (Thermo Fisher) coupled to an Ultimate 3000 RSLC system. After online desalting on a PepMap® precolumn, peptides were separated on a PepMap® C18 column using a 123-min gradient (2%-40% acetonitrile, 0.1% formic acid, 300 nL/min). MS1 scans were acquired in the Orbitrap (375-1,500 m/z, resolution 60,000), followed by MS2 [Top12, higher-energy collision-induced dissociation (HCD) fragmentation, resolution 30,000].

Raw spectra were processed with MaxQuant v2.0.3.0^[50]^ using the Andromeda search engine, with label-free quantification (LFQ), and intensity-based absolute quantification (iBAQ) enabled^[51]^. Spectra were searched against the UniProt *Homo sapiens* reference proteome (release 2023_03), including common contaminants and decoy sequences. Fixed and variable modifications included carbamidomethylation (Cys), oxidation (Met), and N-terminal acetylation. Peptide and protein false discovery rates (FDRs) were set at 1%. Data visualization and statistical analysis were performed using Perseus v1.6.15.0^[52]^.

### RNA extraction, reverse transcription quantitative polymerase chain reaction, and small RNA sequencing

Total small RNA was extracted from EV and WS samples using the miRNeasy Serum/Plasma Kit (Qiagen, #217184) and eluted in 14 µL of RNase-free water. RNA yield was quantified using the Qubit^TM^ microRNA Assay Kit (Thermo Fisher, #Q32881), and size distribution was assessed via the LabChip GX system (Caliper-PerkinElmer) using the Small RNA Assay (#CLS153530) to confirm the presence of miRNAs.

For reverse transcription quantitative polymerase chain reaction (RT-qPCR), 2 ng of RNA was reverse-transcribed using the miRCURY® LNA® RT Kit (Qiagen). qPCR was conducted on 20 pg of complementary DNA (cDNA) using miRNA-specific primers [Supplementary Table 1] and the miRCURY® LNA® SYBR® Green PCR Kit (Qiagen).

Small RNA libraries were prepared from 10 ng of miRNA using the TruSeq Small RNA Library Prep Kit (Illumina, #RS-200-0012). Following adapter ligation, cDNA synthesis, and PCR amplification (11 cycles), libraries were purified with Ampure XP beads (Beckman Coulter, #A63880). Library quality was assessed using the LabChip GX system, and quantification was performed using the Qubit^TM^ dsDNA HS (High Sensitivity) Assay (#Q32851). Equimolar pooling was followed by sequencing on the NextSeq 500 platform using the High Output Kit v2.5 (1.8 pM, #20024906).

Post-sequencing, quality control (FastQC, multiQC), adapter trimming, and Phred score filtering were applied. Reads were mapped to the human genome (GRCh38.p14) and miRBase v20 using the miRDeep2 pipeline for miRNA identification and quantification.

### Bioinformatics and statistical analysis

Proteomics: LFQ data from MaxQuant (v2.0.3.0) were used to analyze protein abundance across WS, UC-, Q-, and M-derived EVs. Data were filtered to exclude contaminants, reverse hits, single-peptide identifications, and inconsistently quantified proteins. LFQ intensities were log_2_-transformed, and missing values were imputed using a “missing not at random” (MNAR) method [Gaussian distribution, 1.8 standard deviation (SD) shift, width 0.3]. Differential expression was assessed using the limma package (R Bioconductor), applying empirical Bayes statistics. Significance was defined as |log_2_FC| ≥ 1 with FDR-adjusted *P* < 0.05. Venn diagrams were generated using an online tool (https://bioinformatics.psb.ugent.be/webtools/Venn/).

miRNA-seq: Raw sequencing data were processed with FastQC (v0.12.1) and MultiQC (v1.23). Adapter trimming and quality filtering (Phred score > 20) were performed using fastp (v0.23.4). Reads were mapped to GRCh38.p14 and annotated with miRBase v20 using the miRDeep2 pipeline (v0.13). miRNA expression quantification and differential analysis were performed with DESeq2 (v1.42.1)^[53]^, using a Wald test under a negative binomial generalized linear model. Multiple testing correction was applied using the Benjamini-Hochberg method. Normalized counts (counts per million, CPM) were calculated via edgeR (v4.0.16). The raw data have been deposited in the Gene Expression Omnibus [GEO; managed by the National Center for Biotechnology Information (NCBI)]^[54]^ and are accessible under GEO Series accession number GSE312585 (https://www.ncbi.nlm.nih.gov/geo/query/acc.cgi?acc=GSE312585).

Functional Enrichment: Target gene prediction and validation were conducted using the multiMiR R package (v1.24.0). Gene Ontology (GO) and Kyoto Encyclopedia of Genes and Genomes (KEGG) pathway enrichment analyses were performed using clusterProfiler (v4.10.1).

General Statistics: All other statistical analyses were performed in GraphPad Prism v10. Data were tested for normality, and non-parametric tests (Wilcoxon signed-rank, Friedman, or Kruskal-Wallis tests) were applied as appropriate. Dunn’s post hoc test was used for multiple comparisons. Results are reported as mean ± standard error of the mean (SEM), with statistical significance set at *P* < 0.05.

## RESULTS

### Experimental workflow and EV characterization

As described previously^[45]^, unstimulated whole saliva was collected from healthy male volunteers and processed immediately [Figure 1]. After sequential low-speed centrifugation, 500 µL of WS was retained for direct analysis, while 1 mL aliquots were subjected to EV isolation by UC, Q, or M. EV preparations were either analyzed fresh or stored at -80 °C. Characterization included NTA, cryo-EM, protein quantification, and Western blotting.

**Figure 2:**
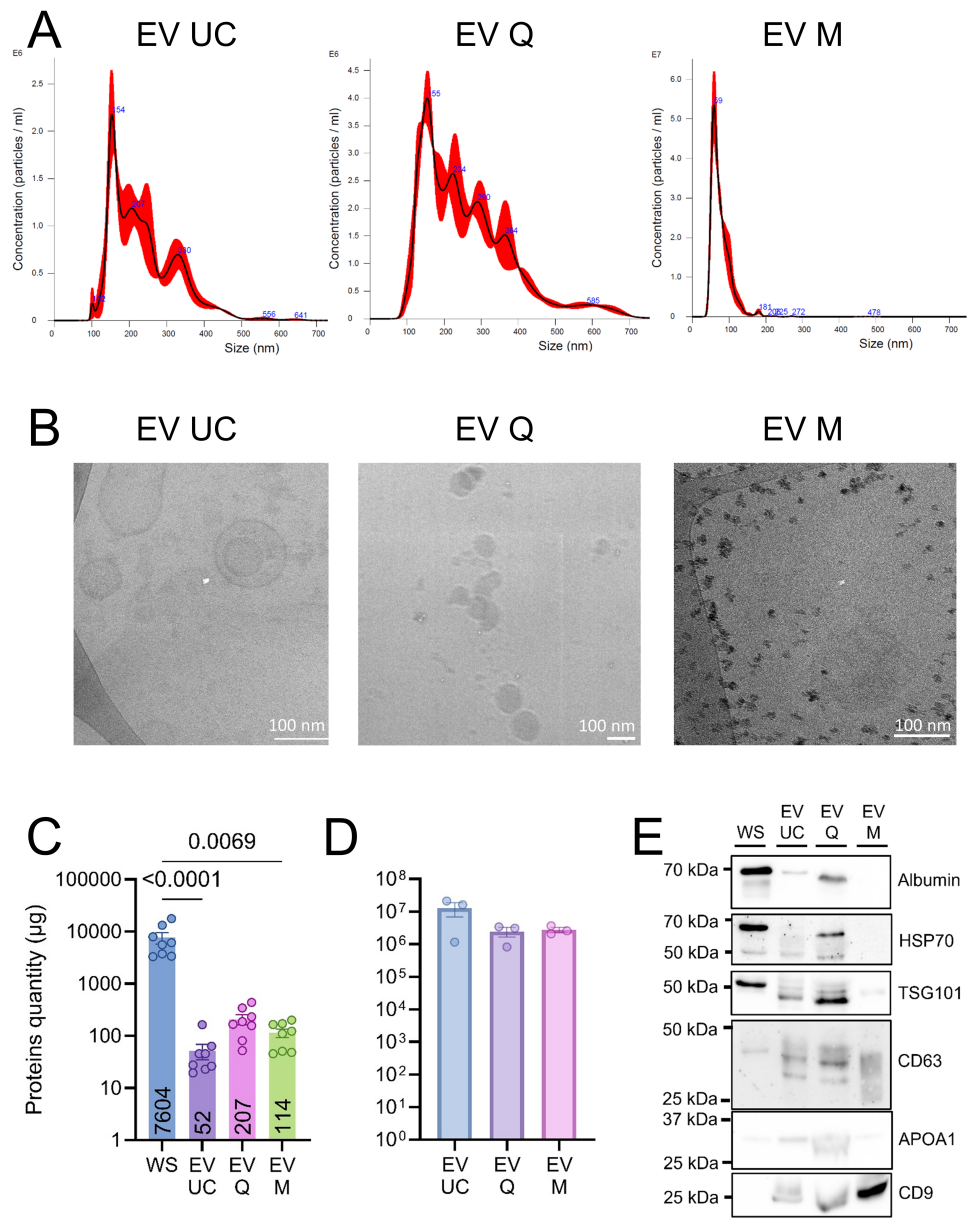
Characterization of human salivary extracellular vesicles. (A) Representative particle size distributions of EVs isolated by UC, Q, and M, as determined by NTA (*n* = 4); (B) Representative cryo-EM images of EVs obtained using each isolation method (UC, Q, M). Scale bars: 100 nm; (C) Mean protein yield (µg ± SEM) obtained from 1 mL of saliva per isolation method, with numerical values indicated below each bar. Statistical analyses were conducted using GraphPad Prism (version 10) with Kruskal-Wallis tests, followed by Dunn’s post hoc test for multiple comparisons. *P* < 0.05 was considered statistically significant; (D) EV purity index (mean ± SEM), calculated as the ratio of particle number to total protein content; (E) Western blot analysis of WS samples and EV fractions isolated by UC, Q, and M. Data represent four independent biological replicates, each assessed in three technical replicates. EV: Extracellular vesicles; UC: ultracentrifugation; Q: PEG-based co-precipitation; M: immunoaffinity capture; WS: whole saliva supernatant; NTA: nanoparticle tracking analysis; SEM: standard error of the mean; HSP70: heat shock protein 70; TSG101: tumor susceptibility gene 101; APOA1: apolipoprotein A1; WB: western blot.

NTA showed that UC and Q yielded EVs with similar size distributions, although Q isolates exhibited a smaller mode diameter (147 nm *vs*. 180 nm) and a lower particle concentration (1.9 × 10^8^ particles/mL *vs*. 4.5 × 10^8^ particles/mL). M-isolated EVs displayed the highest raw particle concentration (1.5 × 10^9^ particles/mL). After subtracting bead-derived background signals, the adjusted particle concentration was 2.85 × 10^8^ particles/mL, with the smallest particle size observed (mean: 134 nm; mode: 108 nm) [Table 1 and Figure 2A]. Cryo-EM confirmed the presence of vesicular structures in all preparations. UC samples occasionally showed vesicle-in-vesicle structures, Q samples exhibited some agglutination with PEG-like residues, and M isolates were well dispersed, although visualization was partially obscured by antibody-coated beads [Figure 2B]. Protein quantification across eight independent experiments (*n* = 8) confirmed effective EV isolation by all three methods. Q samples showed the highest protein quantity (207 µg), resulting in a lower particle-to-protein ratio [Figure 2C]. However, the purity index of Q-isolated EVs remained comparable to those of M and UC, and statistical analysis revealed no significant differences among the three methods [Figure 2D].

**Table 1:** Salivary EV metrics by isolation method

**EV isolation method**	**EV concentration** **(particles/mL ± SD)**	**EV mean size** **(nm ± SD)**	**EV mode size** **(nm ± SD)**	**Protein concentration** **(µg/mL ± SD)**	**Purity**
UC	4.45E+08 ± 2.74E+08	253.3 ± 18.5	180.4 ± 42.8	76.0 ± 76.6	1.87E+07 ± 2.45E+06
Q	1.93E+08 ± 4.94E+07	224.1 ± 15.7	146.6 ± 24.1	106.6 ± 71.0	3.24E+06 ± 1.70E+05
M	2.85E+08 ± 5.24E+08	83.6 ± 10.8	110.0 ± 11.2	106.9 ± 18.6	2.76E+06 ± 3.55E+05

EV: Extracellular vesicles; UC: ultracentrifugation; Q: Qiagen-based precipitation method; M: immunoaffinity-based isolation (Miltenyi Biotec kit); SD: standard deviation.

Western blot analysis confirmed enrichment of EV markers (CD63, CD9, TSG101) in EV preparations compared to WS. Albumin was abundant in WS, faint in UC samples, more pronounced in Q samples, and negligible in M samples, consistent with their respective purity indices. Apolipoprotein A1 (APOA1) was mainly detected in Q samples and, to a lesser extent, in UC and M samples (Figure 2E; uncropped membranes are shown in Supplementary Figure 1).

Overall, all three methods successfully isolated EV-like particles, with distinct profiles in yield, size distribution, and co-isolated proteins [Table 1 and Figure 2].

### Proteomic analysis

**Figure 3:**
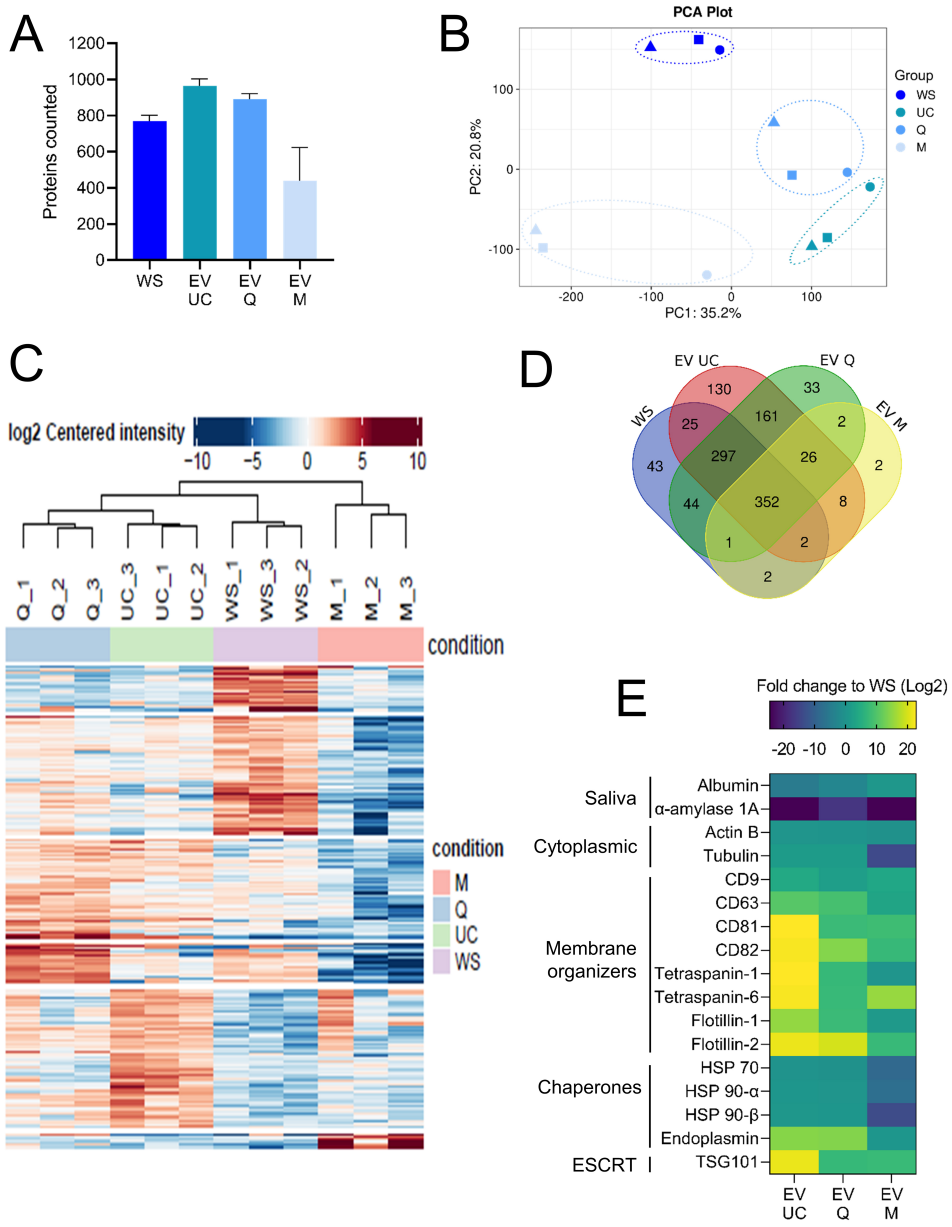
Proteomic analysis of whole saliva and EV fractions. (A) Mean number of proteins identified in WS and in EVs isolated by UC, Q, and M. Proteins were included based on ≥ 2 unique peptides and detection in ≥ 2 of 3 replicates; (B) Principal component analysis of proteomic profiles showing separation between WS and EV samples based on variance in protein expression; (C) Hierarchical clustering heatmap of all samples based on normalized protein abundance; (D) Venn diagram illustrating the overlap of identified proteins among WS (blue), UC- (red), Q- (green), and M- (yellow) derived EVs; (E) Heatmap representing FCs (relative to WS) in UC-, Q-, and M-derived EVs for a subset of proteins commonly reported as salivary or EV-associated markers. WS: Whole saliva supernatant; EV: extracellular vesicles; UC: ultracentrifugation; Q: PEG-based co-precipitation; M: immunoaffinity capture; PCA: principal component analysis; PC1: principal component 1; PC2: principal component 2; HSP: heat shock protein; ESCRT: endosomal sorting complexes required for transport; TSG101: tumor susceptibility gene 101; α-actin: alpha actin; FC: fold change; WS EV: whole saliva EV fraction.

Label-free quantitative proteomics (LC-MS/MS) was performed on WS and EVs isolated by UC, Q, and M (*n* = 3 per method). Using stringent identification criteria (≥ 2 unique peptides, detection in ≥ 2 replicates), we identified an average of 781 proteins in WS, 974 in UC-derived EVs, 902 in Q-derived EVs, and 449 in M-derived EVs [Figure 3A].

Principal component analysis (PCA) indicated that the isolation method was the main source of proteomic variation [Figure 3B]. UC- and Q-derived isolates clustered closely, while M-derived EVs and WS formed distinct groups. Hierarchical clustering of 230 significantly differentially expressed proteins confirmed this pattern [Figure 3C and Supplementary Figure 2]. Across all conditions, 352 proteins were shared between WS and EVs, while 362 were unique to EV preparations. UC and Q shared 161 proteins, compared to only 36 proteins shared between M and the other methods. Both UC and Q also showed broader overlap with WS (297 shared proteins) [Figure 3D].

Comparative abundance analysis revealed consistent enrichment of EV-associated proteins in all EV isolates compared to WS [Figure 3E]. UC and Q showed the strongest enrichment, while M isolates had lower overall protein abundance. Salivary contaminants, such as albumin and α-amylase, were depleted in EV samples relative to WS.

**Figure 4:**
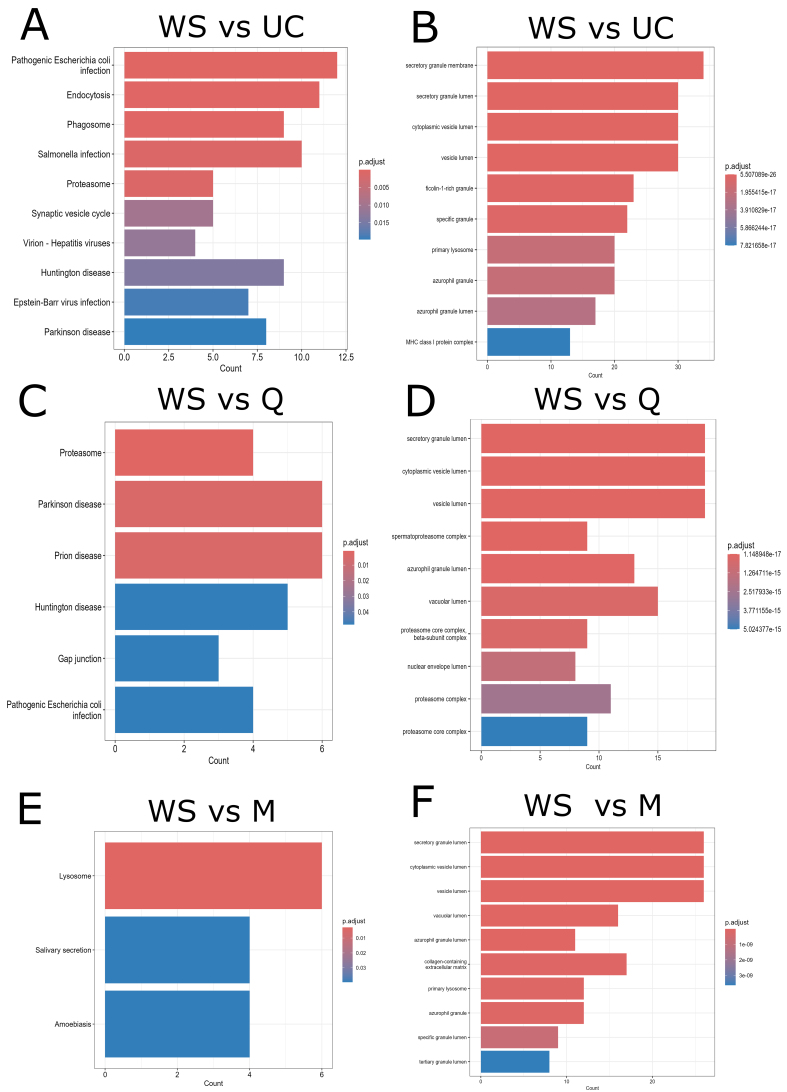
Functional enrichment analysis of differentially abundant proteins between whole saliva and EV fractions. Bar plots illustrate the results of functional enrichment analyses performed using the ClusterProfiler R package (v4.10.1) on proteins differentially abundant between WS and each EV isolation method. (A, C and E) KEGG pathway enrichment for WS *vs*. UC, WS *vs*. Q, and WS *vs*. M, respectively; (B, D and F) GO enrichment analysis (CC category) for WS *vs*. UC, WS *vs*. Q, and WS *vs*. M, respectively. The x-axis indicates the number of proteins associated with each enriched pathway or CC, and the y-axis lists the top enriched terms. Bar color reflects the adjusted *P*-value (Benjamini-Hochberg), with redder colors indicating greater statistical significance. WS: Whole saliva supernatant; EV: extracellular vesicles; UC: ultracentrifugation; Q: PEG-based co-precipitation; M: immunoaffinity capture; KEGG: Kyoto Encyclopedia of Genes and Genomes; GO: Gene Ontology; BP: biological process; CC: cellular component.

Functional enrichment analysis revealed method-specific signatures. KEGG pathway analysis of UC isolates showed enrichment in proteasome-related and intracellular pathways [Figure 4A], while GO Cellular Component (CC) analysis confirmed enrichment of typical EV-associated terms with high statistical significance (adjusted *P*-values ranging from 1e-26 to 1e-17) [Figure 4B].

For Q isolates, KEGG analysis revealed similar enrichment in proteasome-related and intracellular pathways [Figure 4C], and GO CC analysis also confirmed enrichment of EV-associated terms with comparable statistical strength [Figure 4D].

M isolates, analyzed by KEGG, were enriched in lysosomal and vesicle trafficking proteins, as well as salivary secretion components [Figure 4E], while GO CC analysis of M isolates showed enrichment of EV-associated terms with lower statistical significance (~1e-9) [Figure 4F].

### Global transcriptomic profiling of miRNAs

**Figure 5:**
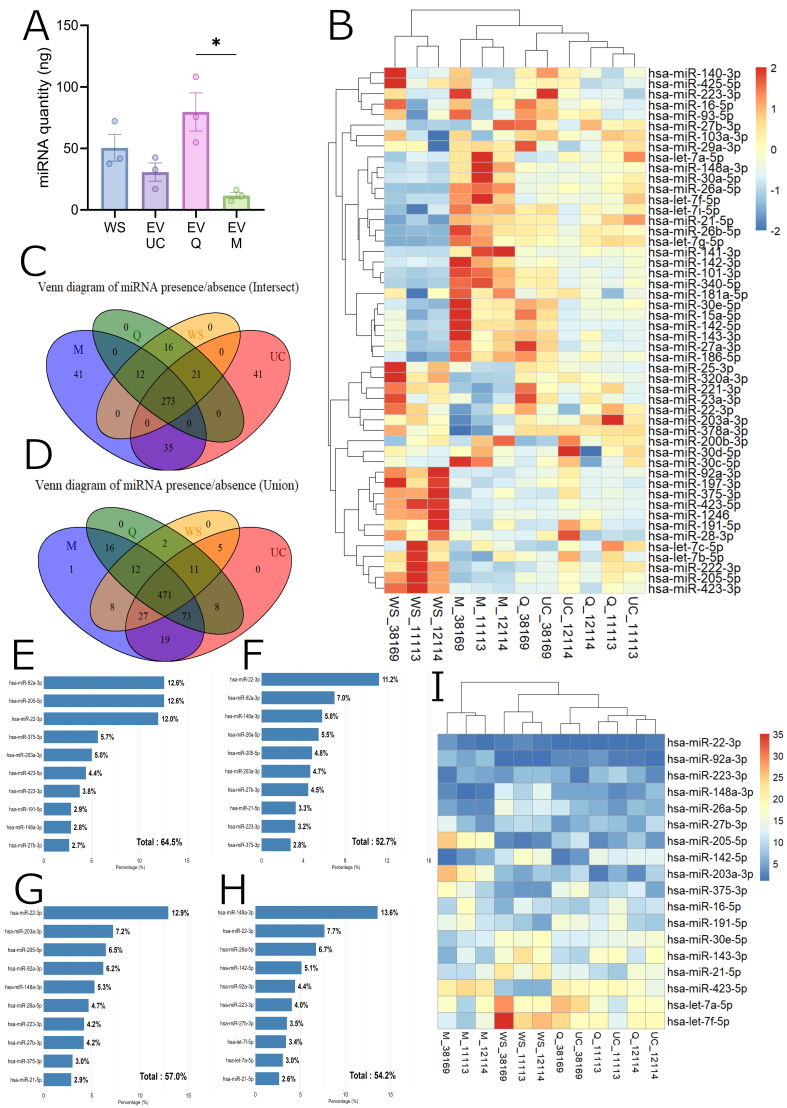
MicroRNA profiling of whole saliva and EV isolates. (A) Total miRNA quantity (mean ± SEM) measured in WS and EV samples isolated by UC, Q, and M. Statistical analyses were performed using GraphPad Prism v10. Kruskal-Wallis tests were applied, followed by Dunn’s post hoc test for multiple comparisons. ^*^P < 0.05; (B) Heatmap of the 50 most abundant miRNAs based on CPM. Values were Z-score normalized (-2 to +2) to highlight relative abundance across conditions (WS, UC, Q, M). The color gradient represents abundance, ranging from low (blue) to high (red); (C and D) Venn diagrams showing the overlap of miRNA species detected in WS, UC, Q, and M. (C) Union: miRNAs detected in at least one replicate per condition. (D) Intersection: miRNAs detected in all three replicates per condition. Colors denote conditions (WS: yellow, UC: red, Q: green, M: purple); (E-H) Bar plots of the 10 most abundant miRNAs in each representative sample (WS_11113, UC_11113, Q_11113, M_11113). Relative abundance is expressed as a percentage of total CPM; (I) Rank-based heatmap of miRNAs appearing among the top 10 positions (by CPM) in at least one sample. The color gradient reflects rank. The x-axis represents individual replicates across conditions; the y-axis lists the top-ranked miRNAs. WS: Whole saliva supernatant; EV: extracellular vesicles; UC: ultracentrifugation; Q: PEG-based co-precipitation; M: immunoaffinity capture; miRNA: microRNA; CPM: counts per million; SEM: standard error of the mean; Z-score: standard score; hsa-miR: Homo sapiens microRNA.

To evaluate the impact of the isolation method on RNA cargo, we performed small RNA sequencing on WS and EVs obtained by UC, Q, and M. Total small RNA was extracted with the Qiagen miRNeasy kit and quantified using a Qubit microRNA assay. M-derived samples yielded lower miRNA amounts compared to UC and Q [Figure 5A]. Sequencing libraries were prepared with the Illumina TruSeq small RNA kit and analyzed on a NextSeq 500. After quality filtering and adapter trimming, only miRNAs detected in all three biological replicates in at least one condition were retained.

Hierarchical clustering of the 50 most abundant miRNAs showed clear differences between WS and EVs [Figure 5B]. WS samples formed a distinct cluster, while EV isolates exhibited closer expression patterns. UC and Q samples clustered by donor, indicating high similarity between these methods, whereas M-derived EVs clustered by isolation method, suggesting a more distinct miRNA cargo profile.

Presence-absence analysis indicated that no miRNAs were uniquely detected in WS, while multiple miRNAs were specific to EV fractions [Figure 5C and D]. The top 10 most abundant miRNAs accounted for approximately 53%-65% of total reads, underscoring the dominance of a few highly expressed species [Figure 5E-H and Supplementary Figure 3]. Among the miRNAs consistently enriched across EV samples were hsa-miR-22-3p, hsa-miR-92a-3p, and hsa-miR-223-3p [Figure 5I].

### Differential expression analysis of miRNAs

To assess how the isolation method influences EV-associated miRNA cargo, we compared the expression profiles of EVs obtained by UC, Q, and M against WS. Only miRNAs detected in all three biological replicates within a given condition were retained, resulting in 731 miRNAs analyzed across comparisons [Supplementary Table 2]

**Figure 6:**
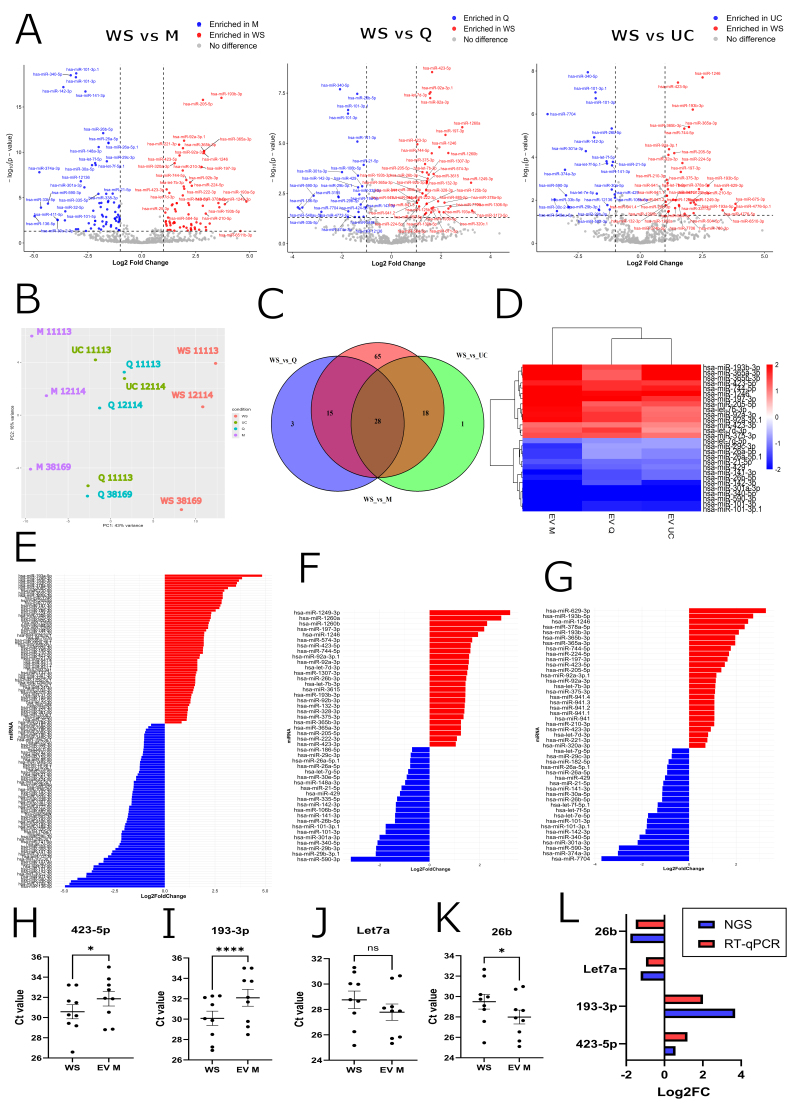
Differential expression analysis of salivary miRNAs across EV isolation methods. (A) Volcano plots showing differentially expressed miRNAs between WS and EV isolates (UC, Q, and M). The x-axis represents log_2_FC, and the y-axis indicates statistical significance as -log_10_(*P*-value). Red dots mark significantly regulated miRNAs (adjusted *P*-value < 0.05; |log_2_FC| > 1). Right shift (positive log_2_FC) indicates upregulation in WS; left shift (negative log_2_FC) indicates enrichment in EVs. Grey dots represent non-significant changes. Analyses were performed using DESeq2 (v1.42.1) and visualized with ggplot2 (v3.5.1); (B) Principal component analysis of global miRNA profiles from WS and EV isolates (UC, Q, M), each with three biological replicates. PC1 and PC2 account for 43% and 16% of the total variance, respectively. Analysis was performed using DESeq2; (C) Venn diagram of differentially expressed miRNAs (adjusted *P* < 0.05) in WS *vs*. UC (green), WS *vs*. Q (purple), and WS *vs*. M (red). Overlaps represent commonly regulated miRNAs across methods. The diagram was generated using the R package VennDiagram (v1.7.3); (D) Heatmap of log_2_FCs for 28 miRNAs significantly and commonly regulated across all three comparisons (WS *vs*. UC, Q, M). Rows represent miRNAs, and columns represent comparisons. The color gradient ranges from red (upregulated in WS) to blue (upregulated in EVs). The heatmap was created using *pheatmap* (v1.0.12), with log_2_FC values scaled from -2 to +2; (E-G) Bar plots showing log_2_FCs of all significantly regulated miRNAs in: (E) WS *vs*. M (*n* = 47 miRNAs), (F) WS *vs*. Q (*n* = 46 miRNAs), (G) WS *vs*. UC (*n* = 128 miRNAs). Red bars indicate miRNAs enriched in WS; blue bars indicate those enriched in EVs. Bar plots were created using ggplot2 (v3.5.1); (H-K) Ct values of miRNAs in WS compared to M samples measured by RT-qPCR. miRNAs including hsa-423-5p (H), hsa-193-3p (I), hsa-let7a (J), and hsa-26b (K) are represented with mean ± SEM (*n* = 9). Statistical analysis was conducted using paired *t*-tests. ^*^P < 0.05 ; ^****^P < 0.0001; (L) Comparison of Log_2_FCs calculated from NGS and RT-qPCR datasets. WS: Whole saliva supernatant; EV: extracellular vesicles; UC: ultracentrifugation; Q: PEG-based co-precipitation; M: immunoaffinity capture; miRNA: microRNA; log_2_FC: log_2_ fold change; PCA: principal component analysis; PC1: principal component 1; PC2: principal component 2; DESeq2: differential expression analysis for RNA-seq data; Ct: cycle threshold; RT-qPCR: reverse transcription quantitative polymerase chain reaction; SEM: standard error of the mean; NGS: next-generation sequencing; hsa-miR: Homo sapiens microRNA.

Volcano plots revealed significant differences in miRNA abundance between WS and the EV fractions [Figure 6A]. The M method displayed the broadest range of significantly downregulated miRNAs (log_2_FC < 0), consistent with the selective enrichment of EV-associated miRNA species.

PCA further emphasized the effects of the isolation method [Figure 6B]. M-derived EVs were distinctly separated from WS along PC1 (43% of the total variance), while UC and Q clustered closely, suggesting similar EV-associated miRNA populations. PC2 (16% of the variance) reflected donor-related variation across all groups. These patterns indicate that M yields a distinct miRNA profile, whereas UC and Q capture more heterogeneous populations.

DESeq2 analysis (FDR < 0.05) identified 47, 46, and 128 differentially expressed miRNAs in UC, Q, and M, respectively, compared to WS [Supplementary Figure 4]. A Venn diagram revealed that 28 miRNAs were consistently altered across all EV isolation methods, while 65 were unique to M, *vs*. 3 for Q and 1 for UC [Figure 6C]. Figure 6D, which focuses on the 28 common miRNAs, indicates a comparable pattern of expression changes across the three isolation methods.

Heatmaps confirmed these trends, with several miRNAs consistently enriched in EVs (e.g., hsa-let-7f-5p, hsa-let-7a-5p, hsa-miR-21-5p, hsa-miR-143-3p), while others were more abundant in WS (e.g., hsa-miR-423-5p, hsa-miR-375-3p) [Figure 6E-G].

Overall, these results demonstrate that the EV isolation method substantially shapes the detectable miRNA landscape, influencing both the overlap with WS and the recovery of potentially informative EV-specific miRNAs.

### RT-qPCR validation

To validate the next-generation sequencing (NGS)-derived differential expression patterns, RT-qPCR was performed on EVs isolated by the M method, which showed the most distinct miRNA profile and the largest number of uniquely differentially expressed species. Four candidate miRNAs were selected: two enriched in WS (hsa-miR-423-5p, hsa-miR-193a-3p) and two enriched in EVs (hsa-miR-26b-5p, hsa-let-7a-5p), based on NGS fold change values.

RT-qPCR was carried out on nine biological replicates. Despite inter-individual variation, the expression patterns were consistent with the sequencing results: hsa-miR-423-5p and hsa-miR-193a-3p were higher in WS [Figure 6H and I], while hsa-miR-26b-5p and hsa-let-7a-5p were enriched in EVs [Figure 6J and K]. Calculated log_2_FCs (-ΔCt, with WS as reference) confirmed both the direction and magnitude of enrichment observed in the NGS data [Figure 6L]. These results support the robustness of the sequencing dataset and confirm that EV isolation methods influence detectable miRNA abundance. To further validate method-dependent miRNA enrichment patterns, RT-qPCR was performed on an independent set of eight biological replicates, directly comparing the three isolation methods [Supplementary Figure 5]. FC analysis relative to WS (2^-ΔCt^) showed that both hsa-miR-26b-5p and hsa-let-7a-5p were consistently enriched in EVs across all conditions. As anticipated, hsa-miR-423-5p and hsa-miR-193a-3p were higher in WS in the M and UC groups. However, this pattern was not observed with the Q method, where these miRNAs failed to show WS enrichment.

### Functional enrichment analysis of validated and predicted target genes of differentially expressed miRNAs

To explore the potential biological relevance, functional enrichment analysis was performed on the validated and predicted targets of differentially expressed miRNAs. The analyses focused on 28 miRNAs commonly altered across all EV isolation methods and 65 miRNAs uniquely identified in M-derived EVs [Supplementary Table 2].

For the shared set of 28 miRNAs, enrichment analysis identified > 15,000 validated targets and > 14,000 predicted targets, while M-specific miRNAs were associated with slightly larger target pools (> 16,000 validated, > 17,000 predicted).

**Figure 7:**
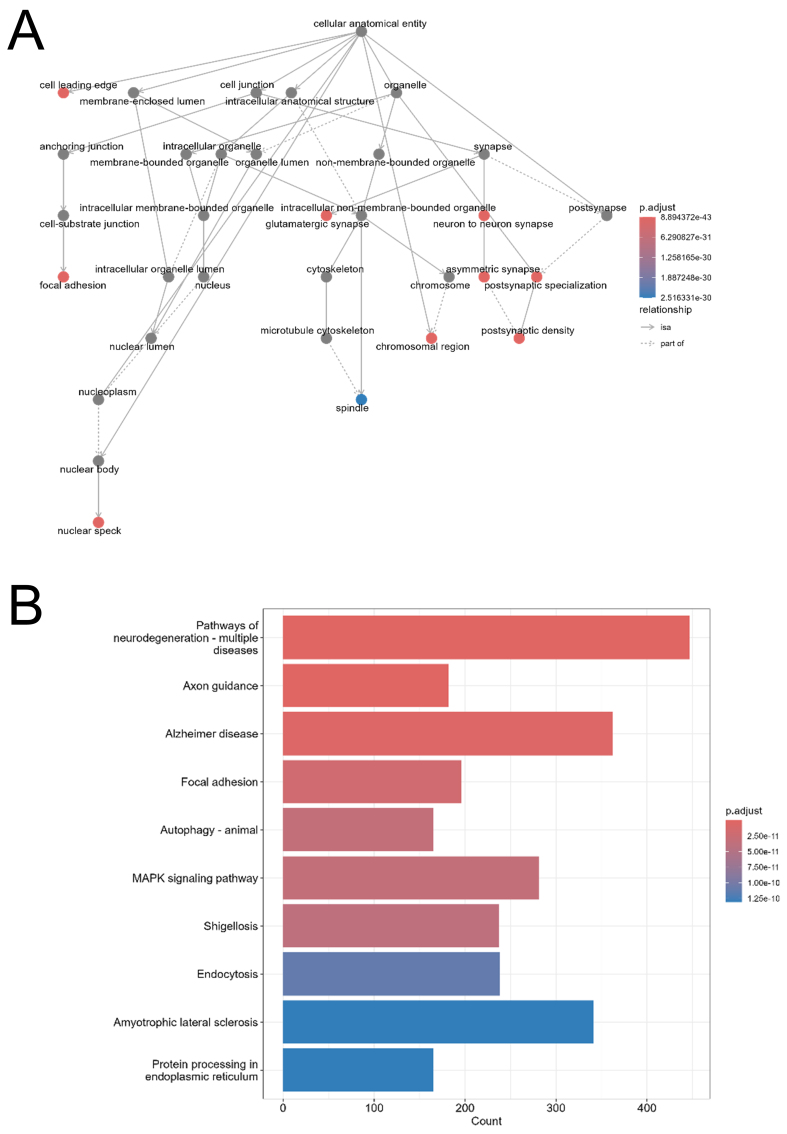
Functional enrichment analysis of target genes regulated by common salivary miRNAs. (A) GO cellular component enrichment network of 15,555 predicted target genes of the 28 miRNAs commonly regulated across all EV isolation methods (WS *vs*. M, Q, UC). Nodes represent GO terms; solid arrows indicate hierarchical subcategories; dashed arrows show associative links. Node color reflects adjusted *P*-value: red for most statistically significant, blue for least statistically significant, and grey for non-significant terms. Analysis and visualization were performed using the clusterProfiler R package (v4.10.1); (B) KEGG pathway enrichment of the same 15,555 target genes. The x-axis shows the number of genes per pathway; the y-axis lists enriched KEGG pathways. The bar color gradient reflects statistical significance (adjusted *P*-value), with red indicating higher significance and blue indicating lower significance. Analysis was conducted with clusterProfiler (v4.10.1) using the KEGG database (https://www.genome.jp/kegg/). WS: Whole saliva supernatant; EV: extracellular vesicles; UC: ultracentrifugation; Q: PEG-based co-precipitation; M: immunoaffinity capture; GO: Gene Ontology; KEGG: Kyoto Encyclopedia of Genes and Genomes; miRNA: microRNA.

GO (CC) analysis indicated consistent associations with neuronal structures, including postsynaptic density, synapses, and axons [Figure 7A, Supplementary Figures 6 and 7]. KEGG pathway analysis highlighted enrichment in axon guidance, autophagy, mitogen-activated protein kinase (MAPK) signaling, and several neurodegeneration-related pathways for the shared miRNAs [Figure 7B]. M-specific miRNAs showed overlapping but broader enrichment patterns, with additional pathways such as cell cycle regulation, focal adhesion, and proteoglycans in cancer [Supplementary Figure 8].

These results indicate that both shared and method-specific salivary EV miRNAs are predicted to regulate genes involved in neuronal and signaling processes. However, the enrichment patterns differ depending on the EV isolation method, reflecting differences in the EV populations captured.

## DISCUSSION

In this study, we conducted a systematic comparison of three widely used EV isolation methods - UC, Q, and M - applied to human saliva. By integrating comprehensive proteomic and transcriptomic analyses, we show that the choice of isolation strategy not only affects vesicle yield and purity, but also strongly influences the qualitative and quantitative composition of salivary EV cargo. These findings emphasize that methodological variability can significantly shape biological readouts and must therefore be carefully considered in biomarker discovery and translational EV research. To support method selection, we summarize the advantages and downstream compatibility of each protocol in Table 2.

**Table 2:** Practical aspects and downstream analysis compatibility of each EV isolation method

	**Ultracentrifugation**	**Co-precipitation** **(PEG-based)**	**Immunoaffinity capture**
**Mechanism of separation**	Size- and density-based separation	Precipitation via surface charge charge modulation using a cationic polymer to co-precipitate EVs	Specific binding of EV surface tetraspanins (CD9, CD63, CD81) using antibody-coated magnetic beads
	Centrifugation at 100,000 × *g*		
**Specificity**	^**^	^*^	^***^
**Convenience/practicability**	^*^	^***^	^**^
**Processing time**	^***^	^*^	^*^
**Hands-on time**	^***^	^*^	^**^
**Cost**	^**^	^*^	^***^
**Scalability**	^*^	^***^	^*^
**Downstream analysis compatibility**			
**NTA**	^***^	^***^	^*^
**Cryo-EM**	^***^	^**^	^*^
**Protein concentration**	^***^	^***^	^**^
**Western blotting**	^***^	^***^	^***^
**RT-qPCR**	^***^	^***^	^***^
**Proteomics**	^***^	^***^	^*^
**NGS**	^***^	^***^	^***^

^*^Low; ^**^moderate; ^***^high. EV: Extracellular vesicles; UC: ultracentrifugation; PEG: polyethylene glycol; NTA: nanoparticle tracking analysis; Cryo-EM: cryogenic electron microscopy; RT-qPCR: reverse transcription quantitative polymerase chain reaction; WB: Western blotting; NGS: next-generation sequencing.

### EV isolation method shapes the proteomic landscape

Our proteomic analysis showed that UC and Q recovered broader and more diverse protein repertoires than M. Despite differences in yield and particle size, UC and Q clustered closely in PCA, indicating that both methods capture a heterogeneous mixture of EV subtypes along with co-isolated non-vesicular proteins. These isolates were enriched in cytosolic proteins, proteasome complexes, and pathways linked to neurodegeneration and intracellular trafficking, consistent with the molecular complexity of saliva. These observations align with previous salivary EV studies, in which broader enrichment strategies improved the detection of low-abundance proteins relevant for biomarker discovery^[26]^. Similar trends have been reported in plasma EV proteomics where UC and precipitation-based methods achieved high proteome coverage but introduced contamination from abundant proteins such as albumin and apolipoproteins^[55,56]^.

In contrast, M, which targets tetraspanins (CD9, CD63, CD81), yielded a more restricted proteome enriched in lysosomal and vesicle trafficking proteins. This pattern suggests the isolation of a narrower small EV subpopulation, likely of endosomal origin and enriched in epithelial- or salivary gland-derived vesicles, consistent with previous descriptions of tetraspanin-positive salivary EVs^[57]^. Although all three methods showed enrichment in canonical EV-associated GO terms, UC and Q showed greater statistical significance, reflecting higher proteomic complexity rather than differences in EV quality^[58]^.

These findings complement recent studies by Kawano *et al*., who described distinct molecular profiles in salivary EV subtypes, and Sandira *et al*., who showed that technical variability introduced by isolation methods can confound biological interpretation^[59,60]^. Collectively, our results highlight a practical dichotomy: UC and Q are better suited for exploratory proteomics and broad-spectrum biomarker discovery, whereas M isolation provides higher purity and reproducibility for targeted applications where background interference must be minimized.

### Method-dependent variation in EV miRNA landscapes

Small RNA sequencing revealed that EV-associated miRNA profiles differ substantially from WS and across isolation methods. As expected, several highly abundant salivary miRNAs were depleted in EV fractions, consistent with previous reports showing that vesicle-encapsulated RNAs represent a selective subset rather than a simple reflection of cell-free biofluid content^[61,62]^.

PCA indicated that the isolation method was the dominant source of variation, exceeding donor-dependent effects. UC and Q samples clustered closely, suggesting that they capture similar vesicle populations, while M-derived EVs formed a distinct cluster. Importantly, this separation reflects the selective enrichment of a specific EV subpopulation by M, rather than improved inter-individual reproducibility. This observation aligns with findings by Buschmann *et al*. in serum, where the isolation strategy strongly influenced small RNA landscapes, particularly for low-abundance species^[63]^.

Differential expression analysis further highlighted method-specific signatures. Twenty-eight miRNAs were consistently altered across all three EV methods compared to WS, suggesting a core EV-associated signature. In contrast, M-derived EVs contained 65 uniquely differentially expressed miRNAs, compared to only a few in UC or Q. This pattern suggests that M facilitates the detection of low-abundance or selectively packaged miRNAs that may be missed by broader enrichment strategies. These observations reinforce our previous findings in saliva^[45]^ and align with studies by Llorens-Revull *et al.* in plasma, where the isolation method determined both the diversity and detectability of miRNAs^[37]^.

To validate these sequencing-derived patterns, we performed RT-qPCR on an independent set of biological replicates, focusing on four miRNA candidates: hsa-miR-26b-5p, hsa-let-7a-5p, hsa-miR-423-5p, and hsa-miR-193a-3p. The RT-qPCR results confirmed the NGS-derived differences, supporting the robustness of our dataset. Specifically, hsa-miR-26b-5p and hsa-let-7a-5p were consistently enriched in EVs across all three isolation methods, reinforcing their association with EV cargo. In contrast, hsa-miR-423-5p and hsa-miR-193a-3p were predominantly retained in WS in the M and UC methods. However, this pattern was not observed with the Q method, where these miRNAs failed to show enrichment in WS. This discrepancy suggests that the Q method may lack specificity, potentially due to co-isolation of non-EV miRNAs or incomplete depletion of saliva contaminants. The UC method, while reliable, appears to offer intermediate sensitivity, making it suitable for applications prioritizing high throughput or cost-effectiveness over maximal specificity.

Importantly, the NGS-derived differences were confirmed by RT-qPCR for selected candidates (e.g., hsa-let-7a-5p, hsa-miR-26b-5p, hsa-miR-423-5p, hsa-miR-193a-3p), supporting the robustness of our dataset and aligning with best practices in EV biomarker studies that emphasize orthogonal validation for translational reliability.

Collectively, these results indicate that while UC and Q provide broader coverage of salivary EV miRNAs, M capture shows a more distinct transcriptomic profile. This trade-off has direct implications for biomarker discovery: broad methods may maximize sensitivity in exploratory studies, whereas selective capture may enhance detection of biologically specialized miRNAs with potential mechanistic or diagnostic value. Ultimately, the choice of isolation method should be guided by study objectives, balancing sensitivity, specificity, and the need for high-resolution miRNA profiling.

### Functional implications: neurodegeneration-linked miRNAs in salivary EVs

Functional enrichment analysis of validated and predicted targets of differentially expressed miRNAs revealed strong associations with neuronal structure and signaling. GO terms such as postsynaptic density, axon, and synapse were consistently overrepresented, highlighting processes central to synaptic plasticity and vulnerable in neurodegenerative diseases. KEGG pathway analysis further identified enrichment in pathways linked to Alzheimer’s disease, amyotrophic lateral sclerosis (ALS), and MAPK signaling. These findings align with previous studies reporting that plasma- and CSF-derived EVs carry neuronally relevant cargo reflective of central nervous system (CNS) pathophysiology^[64,65]^. Our findings extend this concept to saliva, an easily accessible biofluid well-suited for repeated sampling and clinical translational studies.

The 28 miRNAs consistently altered across all EV isolation methods were enriched in neuronal and synaptic pathways, suggesting a conserved salivary EV miRNA signature with potential biomarker relevance. These findings expand on prior work by Rastogi *et al*., who reported that salivary EVs reflect systemic molecular alterations in neurodegenerative contexts, supporting their biomarker potential^[19]^. Notably, the 65 miRNAs uniquely detected in M-derived EVs mapped to additional pathways, including cell cycle regulation, Hippo signaling, and proteoglycans in cancer. These networks are implicated in neural stem cell homeostasis, apoptosis, and neuroinflammation, suggesting that tetraspanin-targeted capture may isolate specialized EV subpopulations, as observed in neural-derived EV studies^[66,67]^.

Beyond neurological pathways, recent studies emphasize the broader biological responsiveness of salivary EV miRNAs. Kazanopoulos *et al.* identified over 1,400 distinct salivary EV miRNAs and demonstrated time-dependent changes during orthodontic treatment, underscoring that salivary EVs can capture dynamic remodeling processes^[68]^. Together with our results, these observations reinforce the potential of salivary EVs as information-rich sources for monitoring both systemic and disease-specific biological states.

Our observations are also consistent with recent translational work. Ryu *et al*. showed that salivary EV-derived miR-485-3p correlated with cerebral amyloid-β burden and predicted positron emission tomography (PET) positivity in patients with Alzheimer’s disease, while Malaguarnera *et al.* further highlighted miR-485-3p among leading salivary EV miRNAs with diagnostic relevance in neurodegenerative diseases, supporting its inclusion in candidate biomarker panels^[17,69]^. Collectively, these studies highlight that salivary EV miRNAs can provide non-invasive readouts of neuronal processes and complement established CSF or plasma biomarkers.

### Strategic recommendations for salivary EV isolation

Our results reinforce that no single isolation method is equivalent; instead, selection should be guided by research objectives and analytical priorities. For exploratory proteomics and discovery-driven studies, UC and Q provide broader coverage of the salivary EV proteome and miRNA repertoire. Their ability to recover heterogeneous vesicle populations increases sensitivity for detecting diverse biomolecules, although this comes at the cost of co-isolating non-vesicular contaminants. For targeted transcriptomics, biomarker validation, or clinical assay development, M offers greater selectivity for CD9/CD63/CD81-positive EVs, reducing background proteins and yielding distinct miRNA signatures. While not necessarily more reproducible across individuals, its specificity makes it suitable when analytical sensitivity and purity are critical. While our study demonstrates the utility of M isolation for capturing small EVs, likely enriched in exosomes, it is important to acknowledge that this method may preferentially isolate specific EV subpopulations. Other EV populations, such as microvesicles, may carry unique disease-associated biomarkers that could be missed when using a size-selective isolation approach. The heterogeneity of EVs in saliva, ranging from exosomes to larger microvesicles, implies that no single isolation method may universally capture all relevant biomarkers. Therefore, the optimal isolation strategy for diagnostic applications in saliva should be empirically determined for each biomarker of interest, ensuring that the chosen method aligns with the specific EV subpopulations most informative for the disease or biological process under investigation. Future studies should systematically compare isolation techniques to identify the most effective approaches for capturing the full spectrum of diagnostically relevant EVs.

In translational pipelines, combining orthogonal approaches or cross-validating results across multiple methods can mitigate isolation-specific biases and strengthen reproducibility. This layered strategy may be particularly valuable when moving from biomarker discovery to clinical validation.

These recommendations align with the MISEV2023 guidelines^[46]^, which emphasize that isolation strategies must be tailored to the biofluid, analyte of interest, and downstream platform. Failure to account for these factors can introduce technical variability that exceeds biological differences, as highlighted by Sandira *et al*.^[60]^. Moving forward, harmonization of protocols and transparent reporting will be essential to enable cross-study comparability and to accelerate the clinical translation of salivary EV biomarkers.

### Study limitations

Several limitations should be acknowledged. First, the omics analyses were conducted using a limited number of biological replicates (*n* = 3), which may restrict statistical power. Larger and more diverse cohorts are needed to validate isolation-specific signatures and to more comprehensively capture inter-individual variability. Second, the functional enrichment analysis relied on computational predictions and validated target databases, both of which require experimental follow-up to confirm biological relevance, particularly for pathways linked to neurodegeneration. Third, size-exclusion chromatography (SEC), a widely used method for EV purification^[70,71]^, was not included in the present study. Although SEC offers high purity, it requires larger input volumes and is sensitive to the viscosity and mucin content of saliva, which can reduce yield and impair column performance^[72,73]^. Therefore, this study focused instead on methods commonly applied in translational workflows (UC, Q, M). Future studies should include SEC and emerging isolation technologies to build a more comprehensive framework for salivary EV research.

### Conclusion

This study provides the first systematic multi-omics comparison of UC, Q, and M for isolating salivary EVs. We demonstrate that the choice of isolation strategy profoundly influences vesicle yield, purity, and molecular cargo, including both proteins and miRNAs. UC and Q recover broader and more heterogeneous proteomic and transcriptomic profiles, making them well-suited for discovery-driven applications. In contrast, M enriches a more selective EV subset characterized by distinct miRNA signatures, some of which are linked to neuronal and neurodegenerative pathways. These findings underscore that the choice of isolation method must be aligned with specific experimental objectives and that method-specific biases should be carefully considered during biomarker development.

### Future directions

Recent advances in the bioengineering and clinical applications of EVs, including those derived from plant and mammalian sources, have underscored their dual potential as innovative drug delivery systems and therapeutic tools^[29]^. To fully realize this potential (particularly in neurodegenerative diseases), future work must prioritize two interconnected strategies: (1) standardization of isolation and characterization protocols to ensure reproducibility across studies; and (2) targeted validation of disease-specific biomarkers in salivary EVs to enhance their clinical utility.

Advanced single-particle characterization approaches, such as nano-flow cytometry, now enable the simultaneous measurement of EV size, concentration, and surface marker expression at the single-particle level, representing an emerging gold standard for EV characterization^[74]^.

Unlike conventional bulk techniques (e.g., NTA, Western blotting), which provide limited resolution of vesicle heterogeneity and cargo distribution, nano-flow cytometry can resolve subpopulations of salivary EVs, including those enriched with neuron-derived markers [e.g., ATPase Na^+^/K^+^ transporting subunit alpha-3 (ATP1A3)] or pathological cargo (e.g., phosphorylated tau, α-synuclein oligomers). Applying this technology to saliva-derived EVs would enable precise quantification of tetraspanin-positive vesicles and lipid bilayer-containing particles [using probes such as CellMask^TM^ Deep Red (CMDR) or ExoBrite dyes], while also facilitating disease-specific biomarker validation. For instance, EVs carrying phosphorylated tau (p-Tau; Thr181), amyloid-beta 42 peptide (Aβ42), or oligomeric α-synuclein have emerged as promising biomarkers for Alzheimer’s and Parkinson’s diseases, detectable years before clinical onset^[75,76]^. Similarly, miRNAs (e.g., miR-132, miR-212) and protein cargo in circulating EVs may reflect early neuronal dysfunction and disease progression^[66,77]^. Implementing these analyses across diverse isolation methods will provide critical quantitative insights into EV purity, composition, and biological relevance, representing key steps toward establishing standardized benchmarks for salivary EV characterization.

Moving forward, harmonization of isolation protocols, implementation of robust quality control metrics, and improvement in automation will be critical for reducing technical variability and enabling reproducibility across studies. Such standardization will accelerate the integration of salivary EVs into biomarker development pipelines, supporting their potential as scalable, non-invasive tools for precision diagnostics, including the monitoring of neurodegenerative diseases.

## DECLARATIONS

### Availability of data and materials

The authors declare that all relevant data are provided within the manuscript and its supporting information files. The sequencing data discussed in this publication have been deposited in NCBI’s Gene Expression Omnibus^[54]^ and are accessible through GEO Series accession number GSE312585 (https://www.ncbi.nlm.nih.gov/geo/query/acc.cgi?acc=GSE312585). Each dataset contains microRNA expression raw data files (FASTQ format), processed data files (raw sequencing read counts), and a metadata spreadsheet containing information on the overall study and individual samples. All data are freely available without restriction.

### Copyright

© The Author(s) 2026.
