# Supplementary material for: Comparison of extracellular vesicle isolation methods reveals method-dependent protein and miRNA profiles in saliva
Source: Extracell Vesicles Circ Nucl Acids. 2026 Jun 10;7(2):878–903. doi: 10.20517/evcna.2025.176 (PMC13367162; doi:10.20517/evcna.2025.176)
Supplement: Supplementary file 1 [file evcna-7-2-878-SupplementaryMaterials.zip › evcna60176-SupplementaryMaterials/evcna60176-SupplementaryMaterials.pdf]

Supplementary Materials

Comparison of extracellular vesicle isolation methods reveals method-dependent protein and miRNA profiles in saliva

Elodie Simphor[1], Jérémy Boulestreau[1], Romane Marchal[1], Thi Nhu Ngoc Van[1,2], Franck Molina[1], Malik Kahli[1]

[1]Sys2Diag UMR9005 CNRS - ALCEN, Cap Gamma, Parc Euromédecine, Montpellier 34184, France.
[2]SkillCell, Montpellier 34184, France.

Correspondence to: Dr. Malik Kahli, Sys2Diag UMR9005 CNRS - ALCEN, Cap Gamma, Parc Euromédecine, Montpellier 34184, France. E-mail: malik.kahli@sys2diag.cnrs.fr

Supplementary Table 1. List of miRCURY LNA miRNA PCR Assay

Name	Sequence	GeneGlobe ID
hsa-miR-423-5p	5'UGAGGGGCAGAGAGCGAGACUUU	YP00205624
hsa-miR-193-3p	5'AACUGGCCUACAAAGUCCCAGU	YP00204591
hsa-miR-26b-5p	5'UUCAAGUAAUUCAGGAUAGGU	YP00204172
hsa-let-7a-5p	5'UGAGGUAGUAGGUUGUAUAGUU	YP00205727

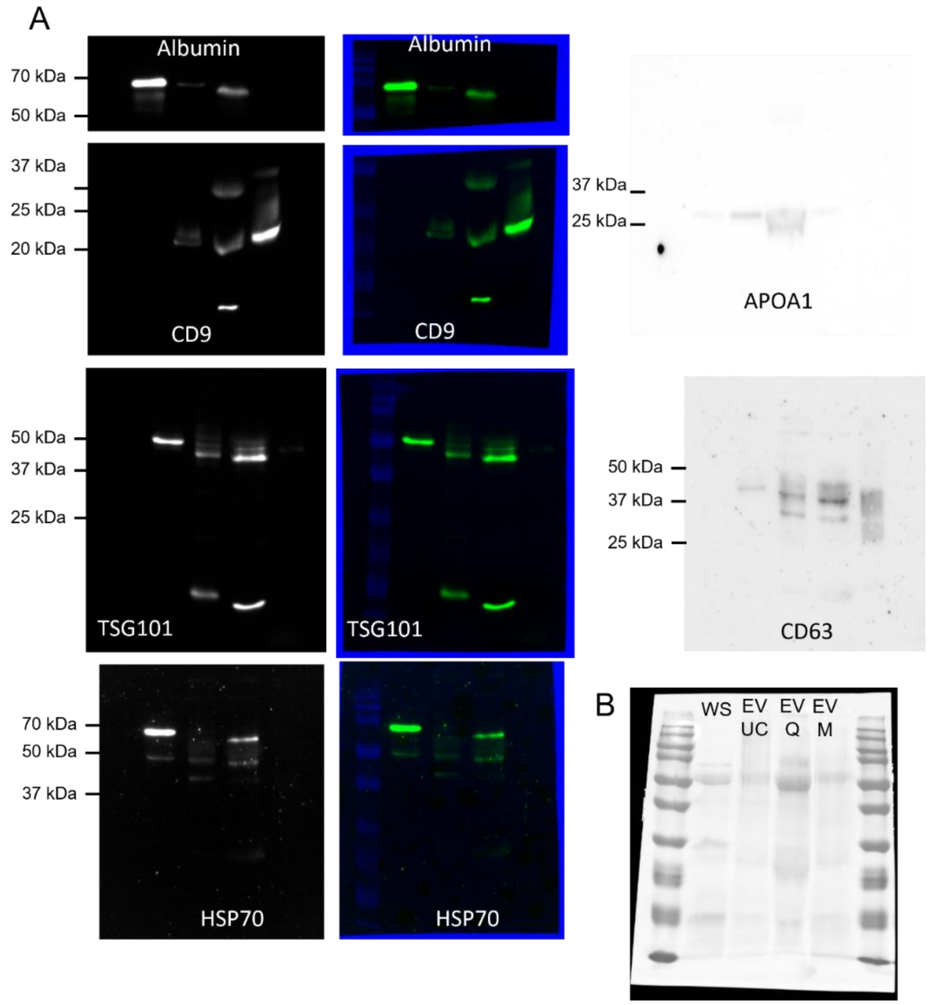

Supplementary Figure 1. Full-length and cut membranes of all immunoblots related to Figure 2E. (A) Uncropped Western blots corresponding to Figure 2E. Molecular Weight are indicated on the left side of the membrane. Some membranes (Albumin and CD9) were cut prior to hybridization with antibodies; (B) Ponceau staining of a representative gel.

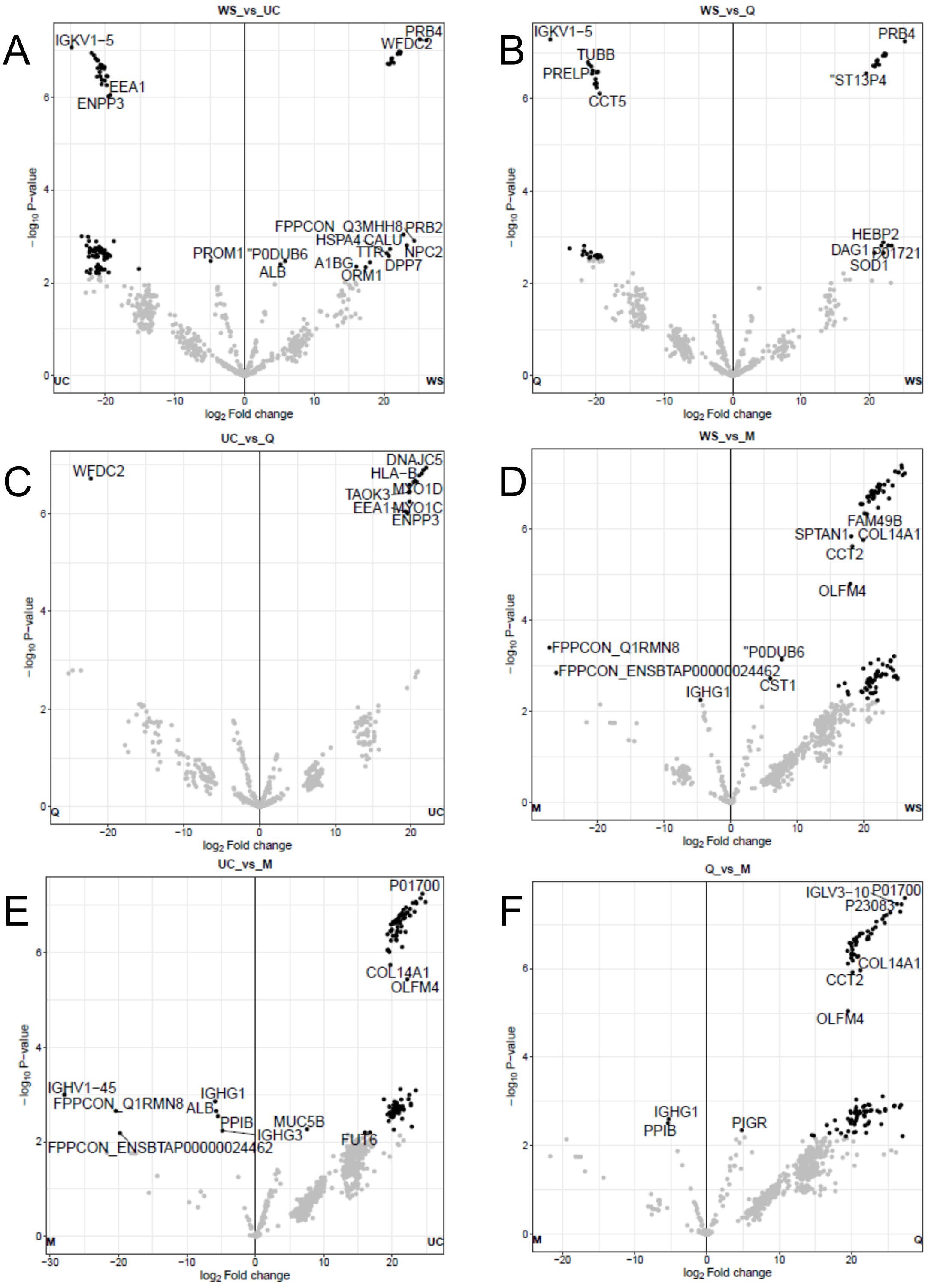

Supplementary Figure 2. Proteomic profiles of salivary EVs and whole saliva. Volcano plots depicting differentially expressed proteins between whole saliva (WS) and salivary extracellular vesicle (EV) isolated by ultracentrifugation (UC), co-precipitation (Q), or immunoaffinity capture (M). Comparisons include: (A) WS vs. UC, (B) WS vs. Q, (C) WS vs. M, (D) UC vs. Q, (E) UC vs. M, and (F) Q vs. M. The x-axis indicates $\log_2$ fold change ($\log_2$FC); the y-axis represents $-\log_{10}$(p-value).

Proteins meeting significance criteria (adjusted p-value < 0.05 and $|\log_2 FC| > 1$) are shown in black. Non-significant proteins are shown in grey. Plots were generated using *LFQ-Analyst*.

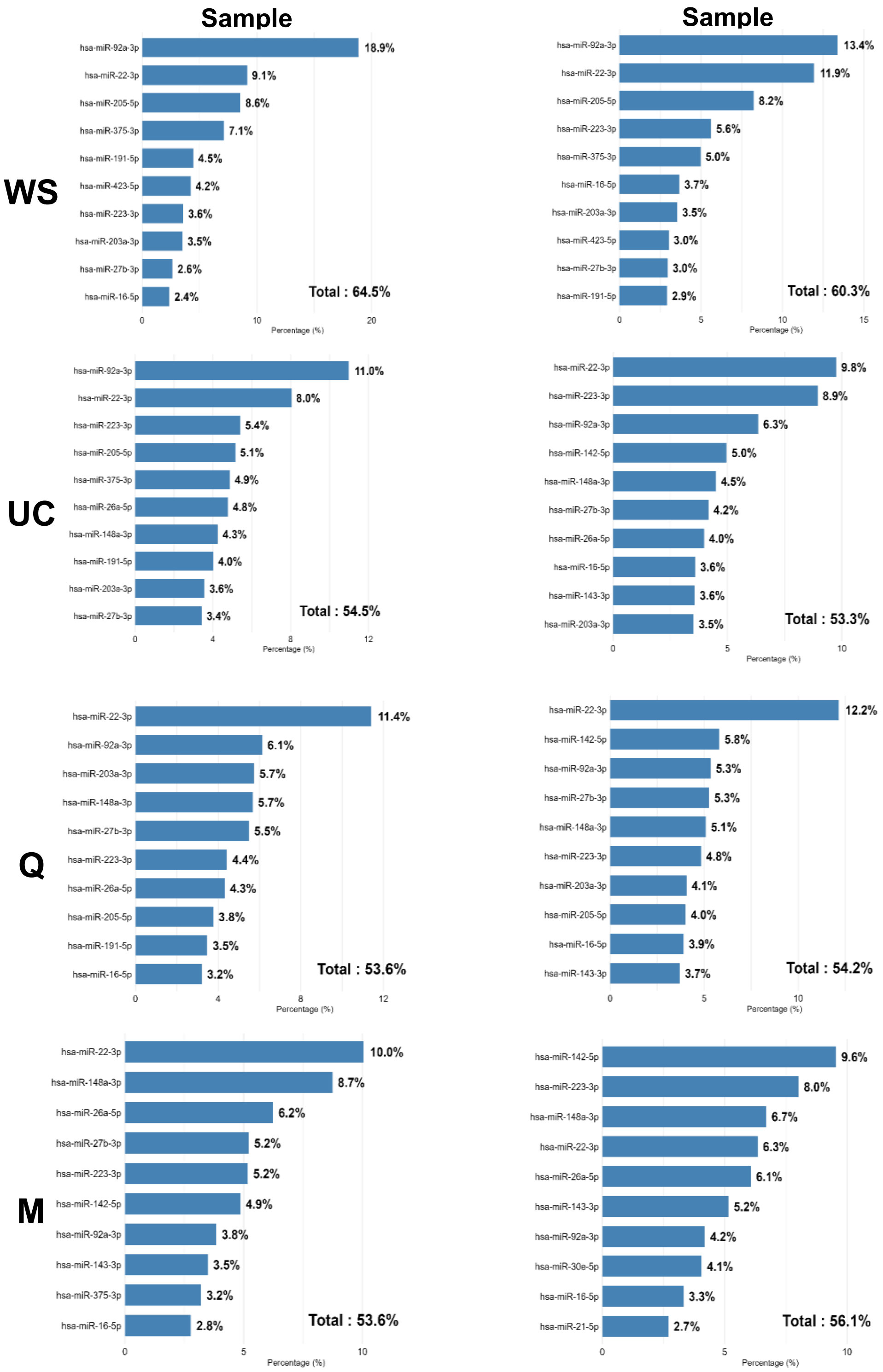

Supplementary Figure 3. Top 10 most abundant miRNAs in individual samples. Bar plots displaying the top 10 most abundant microRNAs (miRNAs) in two individual

donors: sample 12114 (left) and sample 38169 (right), across conditions: whole saliva (WS), ultracentrifugation (UC), co-precipitation (Q), and immunoaffinity (M), respectively. Relative abundance is shown as the percentage of total counts per million (CPM). Graphs were generated using the R package ggplot2 (version 3.5.1).

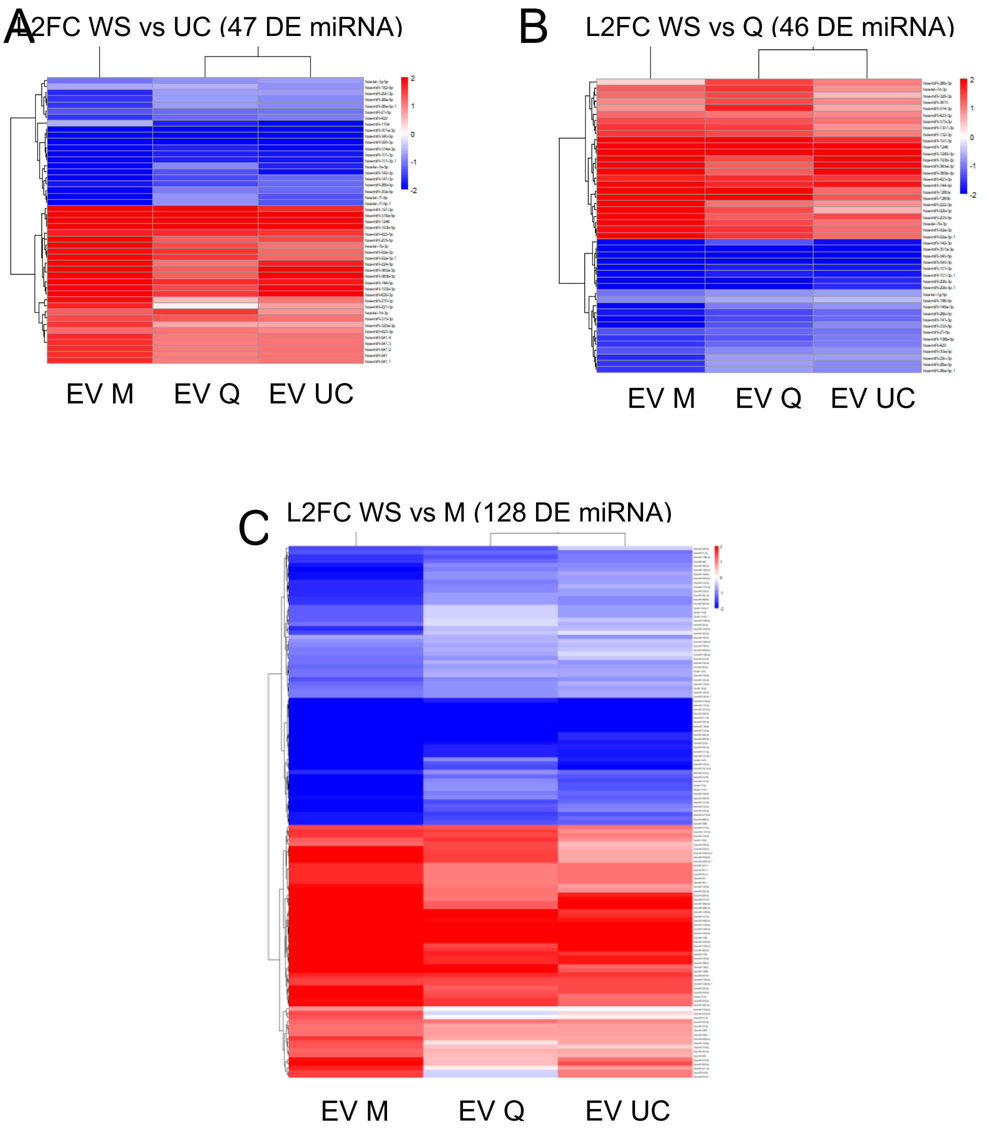

Supplementary Figure 4. Heatmaps of differentially expressed miRNAs between whole saliva and EV isolation methods. Heatmaps showing the $\log_2$ fold change (Log_2FC) of significantly differentially expressed miRNAs (adjusted p-value < 0.05) in the following comparisons: (A) WS vs UC (47 miRNAs), (B) WS vs Q (46 miRNAs),(C) WS vs M (128 miRNAs).Each heatmap represents Log_2FC values normalized on a scale from –2 to +2. The x-axis indicates the comparison between conditions; the y-axis lists differentially expressed miRNAs. Red indicates positive

Log$_2$FC values (upregulated in WS, downregulated in EVs), while blue indicates negative Log$_2$FC values (upregulated in EVs, downregulated in WS). Heatmaps were generated using the pheatmap R package (version 1.0.12).

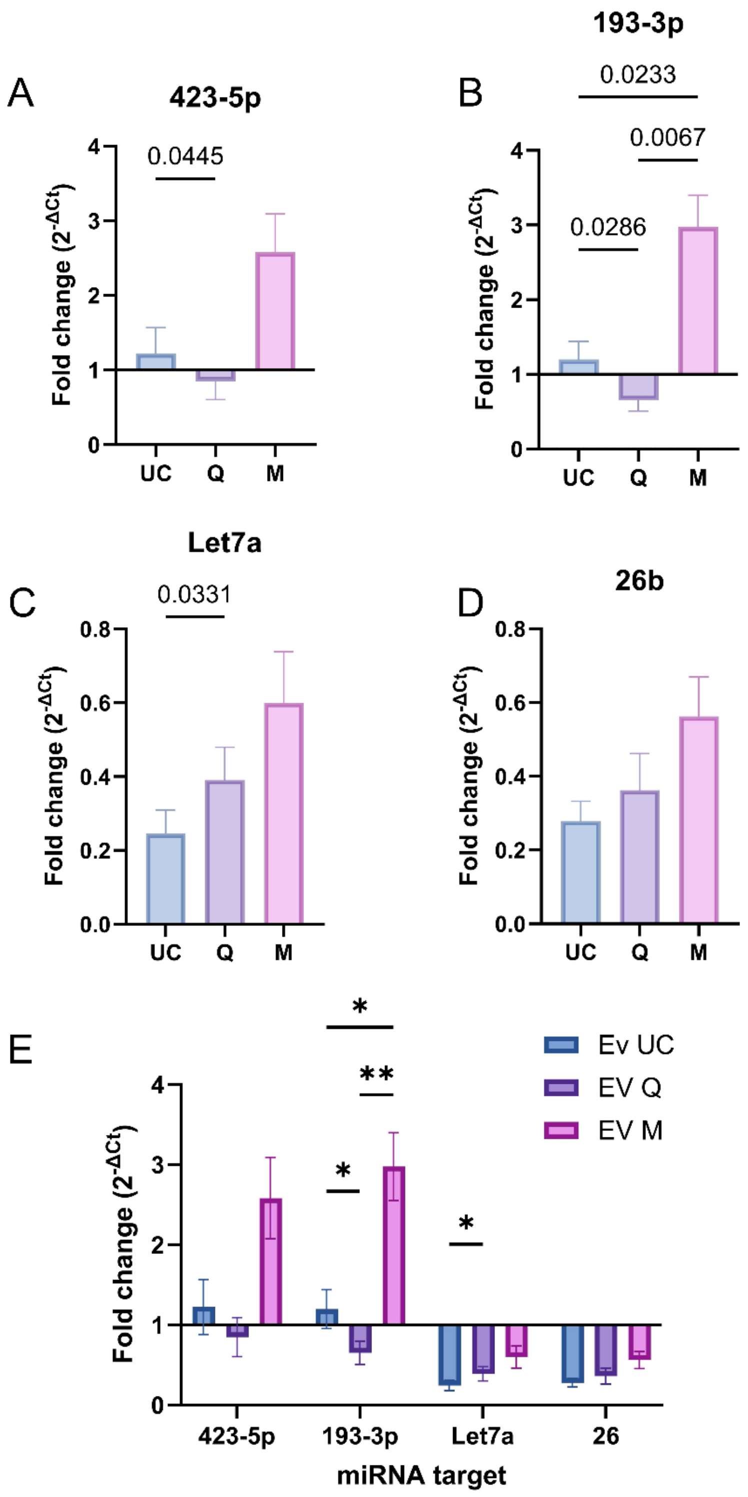

Supplementary Figure 5. Relative expression levels of miRNAs in EVs isolated by three different methods. This figure presents the fold change ($2^{-\Delta Ct}$) of four miRNA

targets in EVs isolated using UC, Q, and M relative to WS. For RT-qPCR, 2 ng of total miRNA were used per sample. (A-D) fold change ($2^{-\Delta Ct}$) of miRNA hsa-423-5p (A), hsa-193-3p (B), hsa-let7a (C) and hsa-26b (D) in EVs (methods: UC, Q, M) compared to WS. Data are presented as mean ± SEM (n=8). Statistical analyses were conducted using one-way ANOVA with Dunn's post-hoc multiple comparison test in GraphPad Prism (version 10). p-values < 0.05 are indicated. (E) Comparative overview of fold change ($2^{-\Delta Ct}$) for all four miRNA targets across the three EV isolation methods relative to WS (baseline set at 1). Each miRNA target is indicated on the x-axis. Statistical significance: *: $p < 0.05$; **: $p < 0.01$.

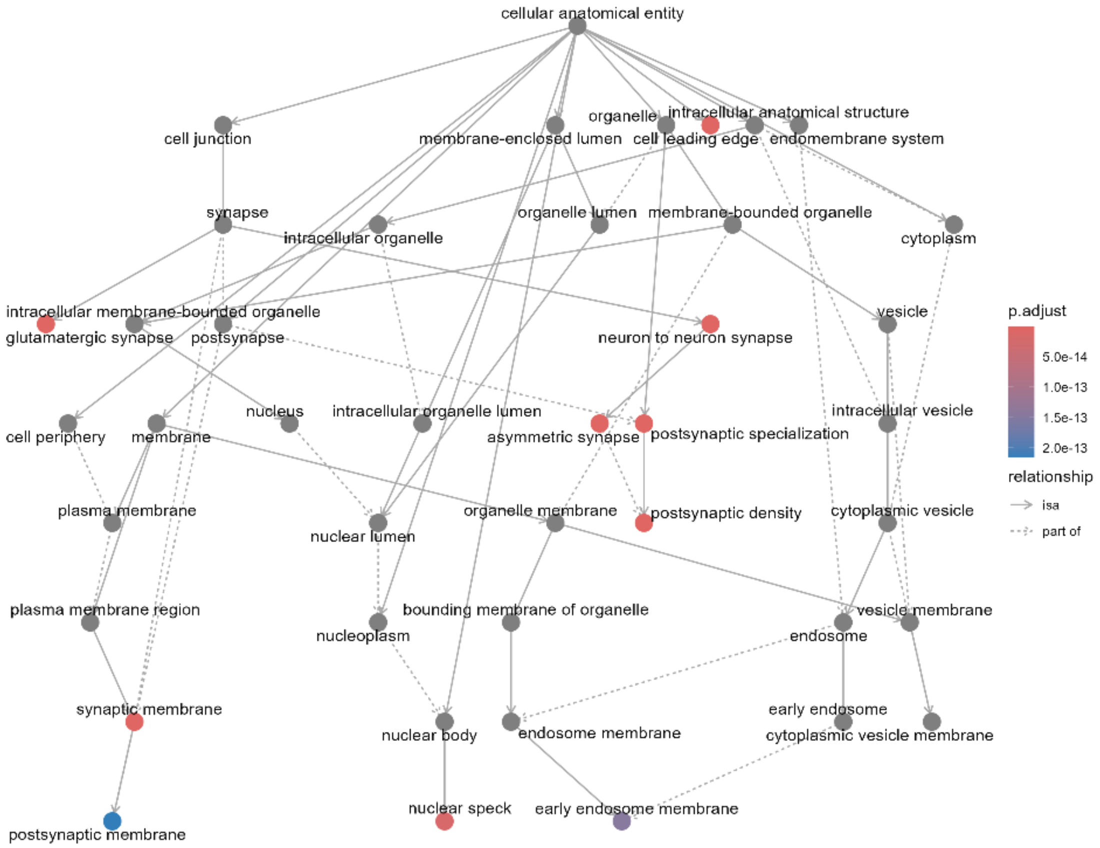

Supplementary Figure 6. Functional enrichment network of predicted gene targets in the Gene Ontology Cellular Component category. Network graph showing Gene Ontology (GO) Cellular Component (CC) enrichment based on 14,635 predicted target genes of the 28 common and differentially expressed miRNAs identified in WS vs M, WS vs Q, and WS vs UC comparisons. Nodes represent enriched GO terms; solid arrows indicate hierarchical subcategories, and dashed arrows show broader category relationships. Red nodes: GO terms with low adjusted p-values (high significance). Blue nodes: GO terms with higher adjusted p-values (lower significance). Grey nodes:

non-significant terms. The analysis was performed using the clusterProfiler R package (version 4.10.1).

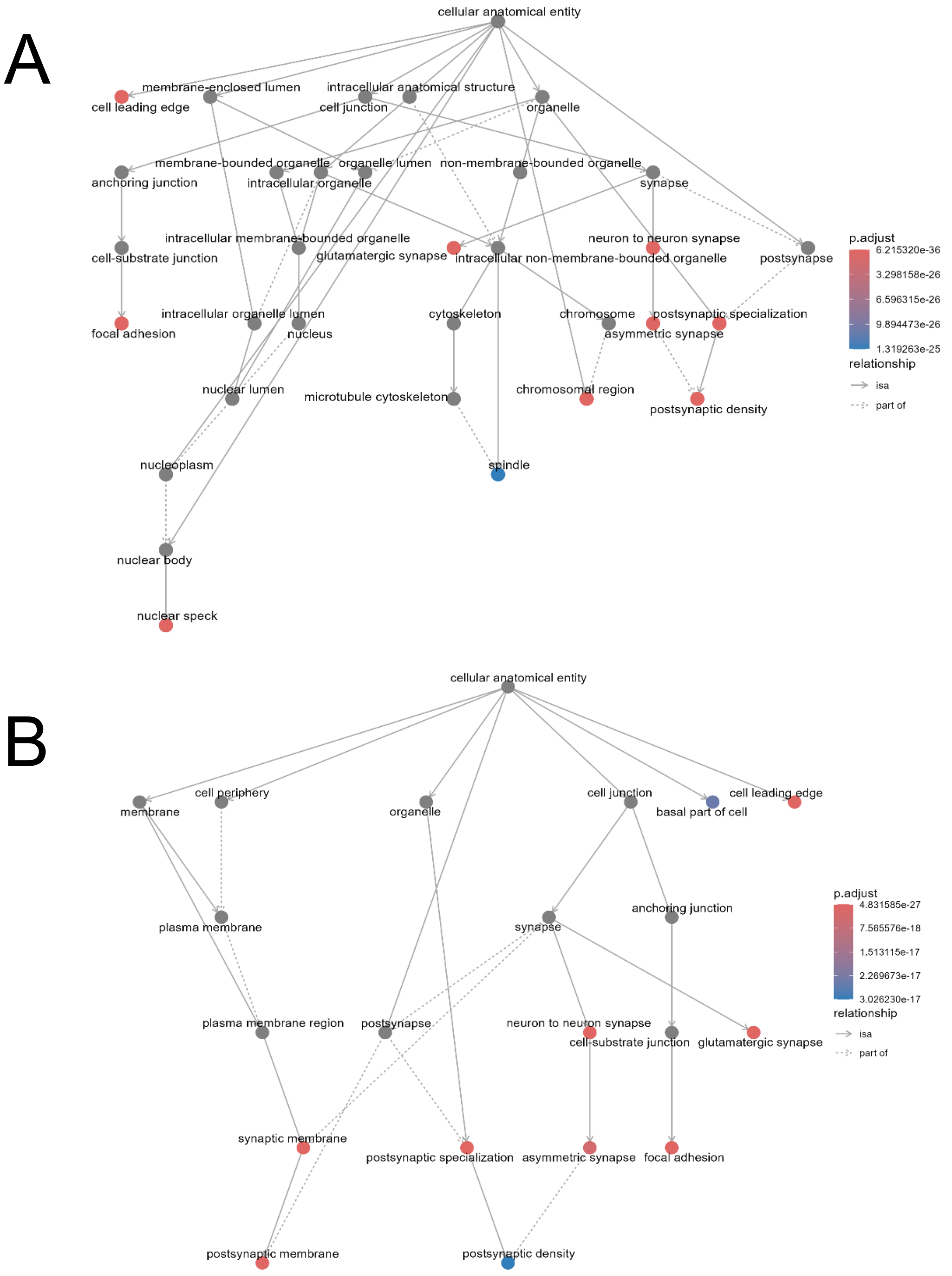

Supplementary Figure 7. Functional enrichment of gene targets of miRNAs differentially expressed in WS vs M. (A) Network graph of Gene Ontology (GO) Cellular Component (CC) terms enriched among 16,397 validated target genes of the 65 miRNAs significantly differentially expressed between WS and M. (B) Network graph of GO-CC terms enriched among 17,674 predicted target genes of the same 65

miRNAs. Nodes represent enriched GO categories. Solid arrows indicate hierarchical subcategories. Dashed arrows show relationships between parent categories. Red nodes: highly significant GO terms (low adjusted p-values). Blue nodes: less significant GO terms (higher adjusted p-values). Grey nodes: non-significant terms. Functional enrichment analyses were performed using the clusterProfiler R package (version 4.10.1).

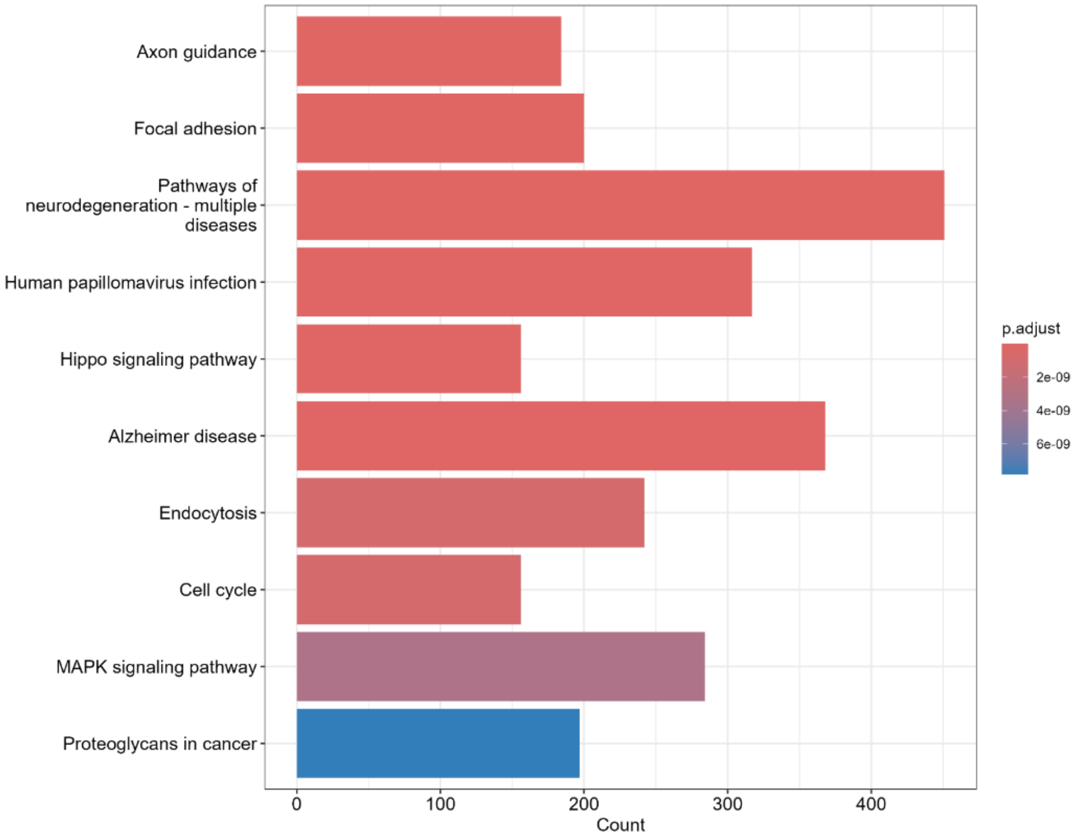

Supplementary Figure 8. KEGG pathway enrichment of validated target genes of M-specific miRNAs. Bar chart showing KEGG pathway enrichment for 16,397 validated target genes of the 65 miRNAs uniquely differentially expressed in the M (immunoaffinity) condition. The x-axis indicates the number of genes associated with each pathway, while the y-axis lists the enriched KEGG pathways. The color gradient represents adjusted p-values: red indicates higher significance (low p-adjusted value), and blue indicates lower significance. Functional enrichment analysis was performed using the clusterProfiler R package (version 4.10.1) with the KEGG database (https://www.genome.jp/kegg/).
